# Supplementary material for: Shibboleth: An agent-based model of signalling mimicry
Source: PLoS One. 2023 Jul 31;18(7):e0289333. doi: 10.1371/journal.pone.0289333 (PMC10389733; doi:10.1371/journal.pone.0289333)
Supplement: S1 File — (ZIP) [file pone.0289333.s001.zip › Supporting-information/Supporting information.docx]

# Supplement — model description

This description follows the standardized ODD protocol for agent-based models (ABMs; Grimm et al. 2006).

*Purpose*

We developed an ABM to simulate the interaction described in the Biblical story of Shibboleth. The aim of the model is to determine whether variables representing cost or risk of detection are a better predictor of which trait in a given set of traits will go into fixation in two repeatedly interacting populations.

*Assumptions*

Our models rely on several theoretical assumptions give in **S1 Table**. See main text for details.

| Assumption | Justification |
| --- | --- |
| Individuals cooperate with others bearing the same or similar signal | Parochial altruism theory (see Fu et al., 2012, *inter alia*) |
| Cultural traits may drive cooperative preference | Cultural group selection theory (see Henrich and Henrich, 2007; *inter alia*) |
| Quality of mimicry detection is frequency-dependent | Hypotheses around competitive coevolutionary relationships (eg, the Red Queen hypothesis, Van Valen, 1973) suggest that receivers must improve detection ability to keep mimics in check |
| Quality of mimicry mirrors quality of mimicry detection | Terms are relative and therefore necessarily coupled |
| Higher quality mimicry and mimicry detection are more costly | Standard signalling theory (ie, Zahavi’s 1975 concept of the handicap principle); here, high-quality traits are costlier for individuals |

**S1 Table: Theoretical assumptions of the Shibboleth models.**

*Parameters and schedule*

| Variable | Type | Description |
| --- | --- | --- |
| N | Global | Number of individuals in blue and green groups; total population = 2 * N; default = 50 |
| generations | Global | Number of generations per model run; default = 200 |
| cost | Global | Cost individual pays on detection; range 0-100; default = 10; coupled in models 1, 2, and 4, decoupled in 3 (see main text) |
| B | Global | Variable indicating the global boundaries of mimicry and sensitivity; where 0 is lowest and 1 is highest; range 0-1; default = 0.2 |
| Traits | Global | Number of signals on blue-green spectrum that individuals may possess; each individual initiates with only 1 trait; default = 10 |
| Mimicry boundaries | Global | Boundaries of possible agent-level mimicry score, determined initially by (traits-1) * B. |
| Tolerance boundaries | Global | Boundaries of possible agent-level sensitivity score, determined initially by (traits-1) * B. |
| Adjustment | Global | Determines tolerance boundary adjustment at end of each generation. Globally defined as round((traits-1)*B). Adjustment is negative if mimics make up at least 50% of population; adjustment is negative if mimics make up less than 50% population. |
| Potential to reproduce (PTR) | Agent | Probability that a given individual will reproduce; individuals initiate with random normal PTR around 50; range 0-100 |
| Mimicry | Agent | An individual’s ability to mimic signals, where 0 is lowest and mimicry boundaries is highest. Individuals initiate with random mimicry sampling from mimicry boundaries. |
| Sensitivity | Agent | An individual’s ability to detect mimicry, where 0 is lowest and tolerance boundaries is highest. Individuals initiate with random sampling from tolerance boundaries. |

**S2 Table: Overview of agent- and global-level variables in the models.**

We represent time discreetly over generations; we do not consider space. At each generation, agents follow the commands described in the schedule (**S1 Fig**).


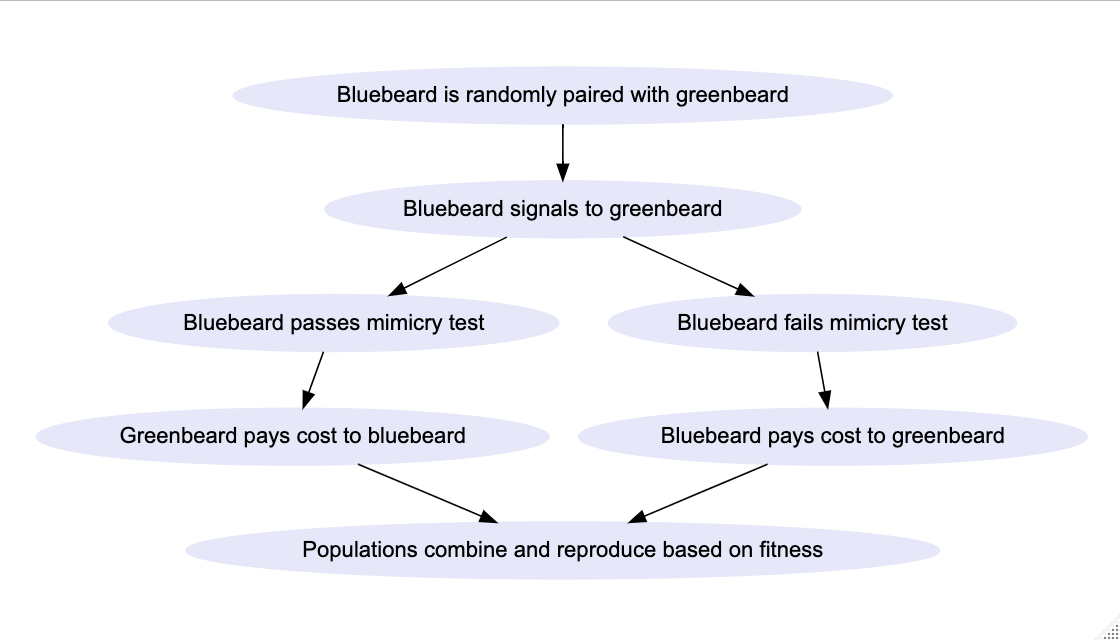


**S1 Fig: Schedule overview; default for all models.**

*Schedule overview for all models*

1. Random dyads form (agent from blue group approaches agent from green group)
2. Agent from blue group requests to enter group and to receive help
3. Agent from green group initiates detection test
   1. If agent from blue group passes test, green agent pays cost
   2. If agent from blue group fails test, blue agent pays cost
4. Tolerance boundaries adjusted by adjustment (see **S1 Table**)
   1. If mimics make up at least 50% of total population, tolerance boundaries are lowered by adjustment
   2. If mimics make up less than 50% of total population, tolerance boundaries are raised by adjustment.
5. Entire population reproduces with probability PTR

*Design concepts*

*Emergence —* the relationship between cost and risk of detection emerges from interactions between agents.

*Prediction* — agents have no memories and the interactions are considered as standalone events in a given cultural or genetic generation.

*Sensing* *vs signalling* — green agents have sensory apparatuses; blue agents can only signal

*Interaction* — dyadic interactions are formed randomly; blue agents attempt to trick green agents into providing aid

*Stochasticity* — dyadic interactions are random; PTR is probabilistic

*Observation* — we report data per model over 10,500 runs exploring variations in the cost and B variables; we compare results with a stochastic drift model without selection that we ran 5,000 times

*Input* — we assumed a scenario where blue agents asked green individuals for aid, and green individuals attempted to determine whether the signal indicating need came from a true group member. We also assumed that tolerance boundaries would be frequency-dependent, and where mimics made up less than half the total population, tolerance would become more relaxed; we assumed the opposite scenario where mimics made up at least 50% of the population.

# Supplementary plots

*Model 1*

**^
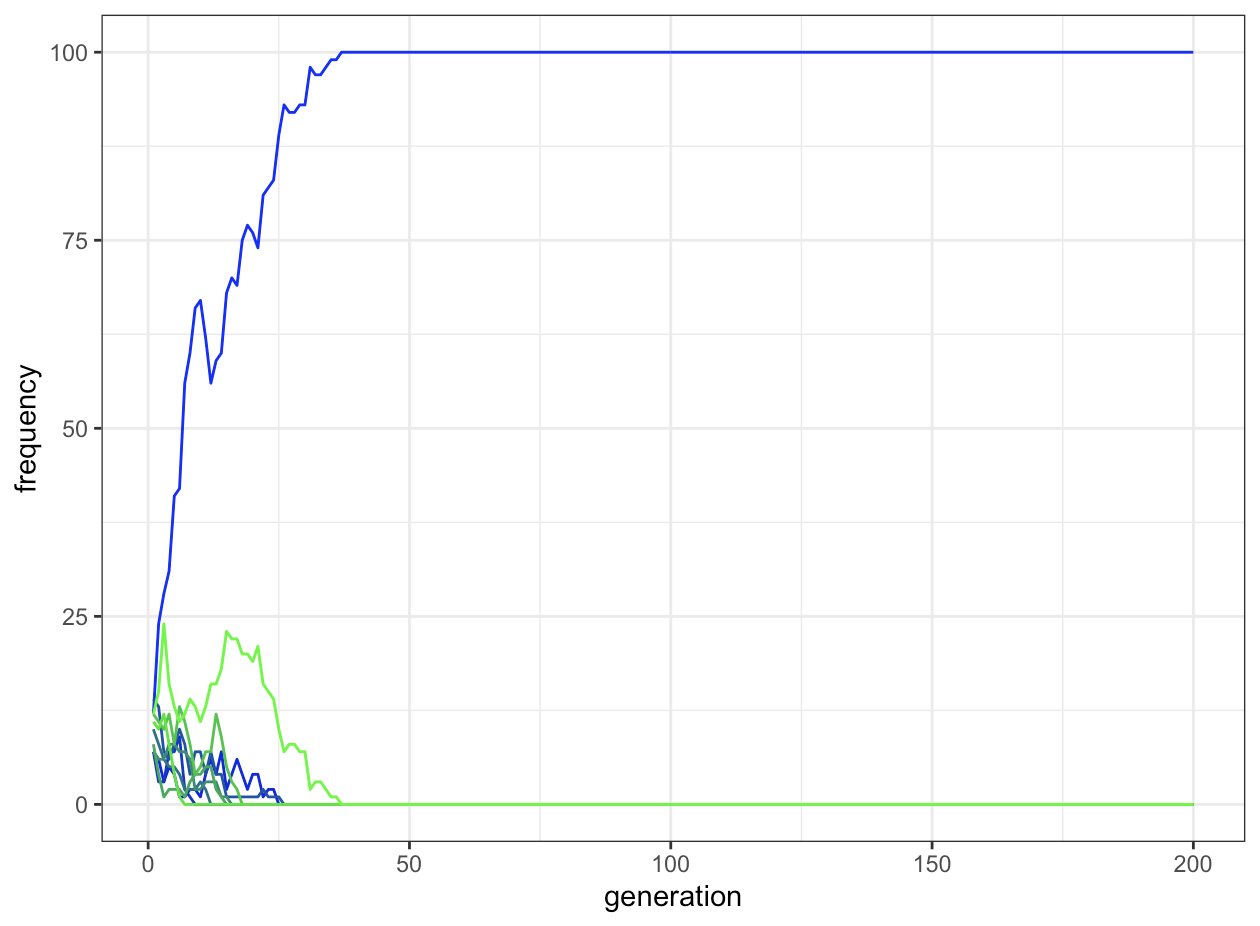
^**

**^S2 Fig. Sample run from drift model.^** ^x-axis = generations over which individuals reproduced; y-axis = number of individuals with a given trait, represented by colours on the blue-green spectrum, in a population of 100 agents (N = 50; generations = 200).^

**^
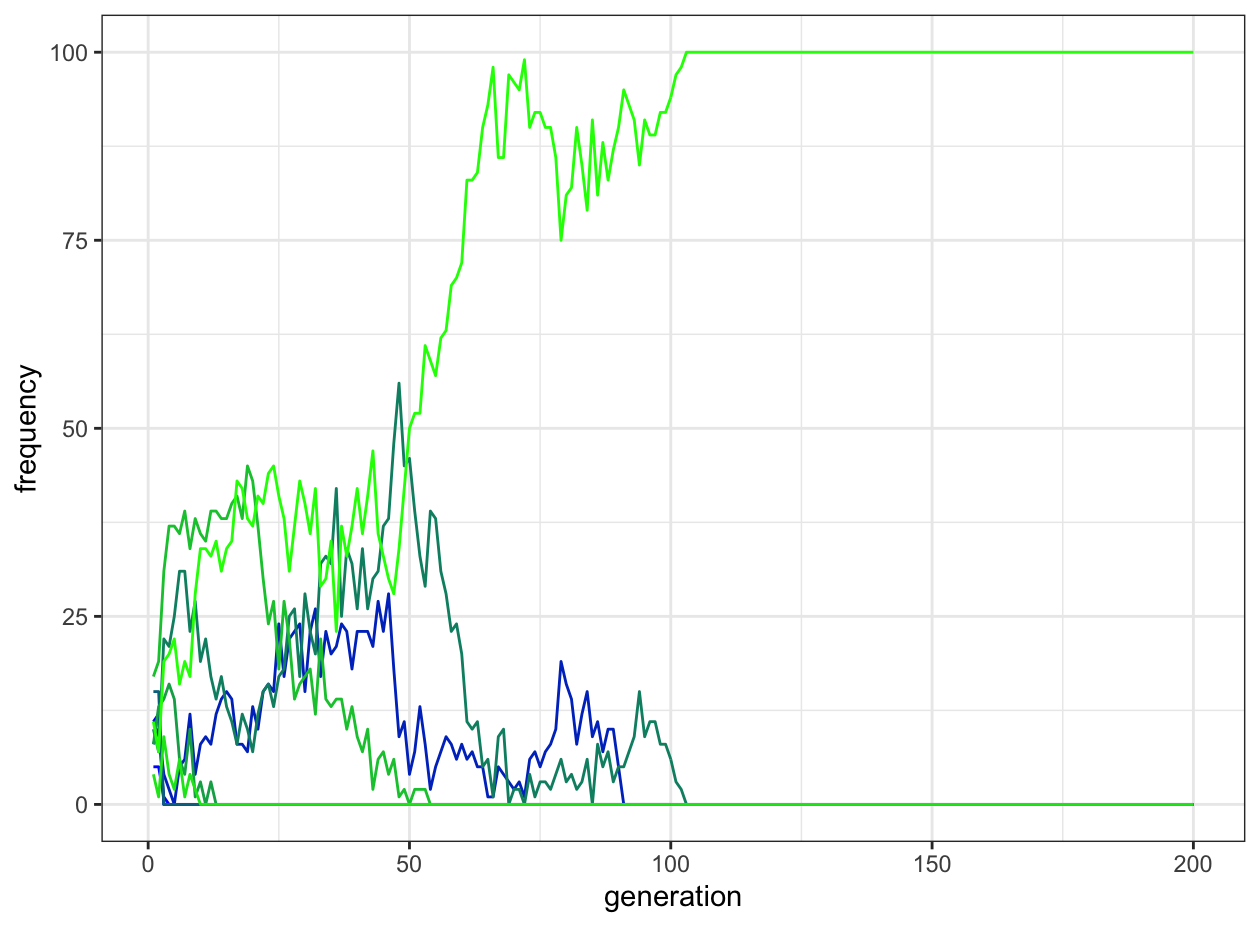
^**

**^S3 Fig. Sample run from model 1 where B = 0 and cost = 10.^** ^x-axis = generations over which individuals reproduced; y-axis = number of individuals with a given trait, represented by colours on the blue-green spectrum, in a population of 100 agents.^


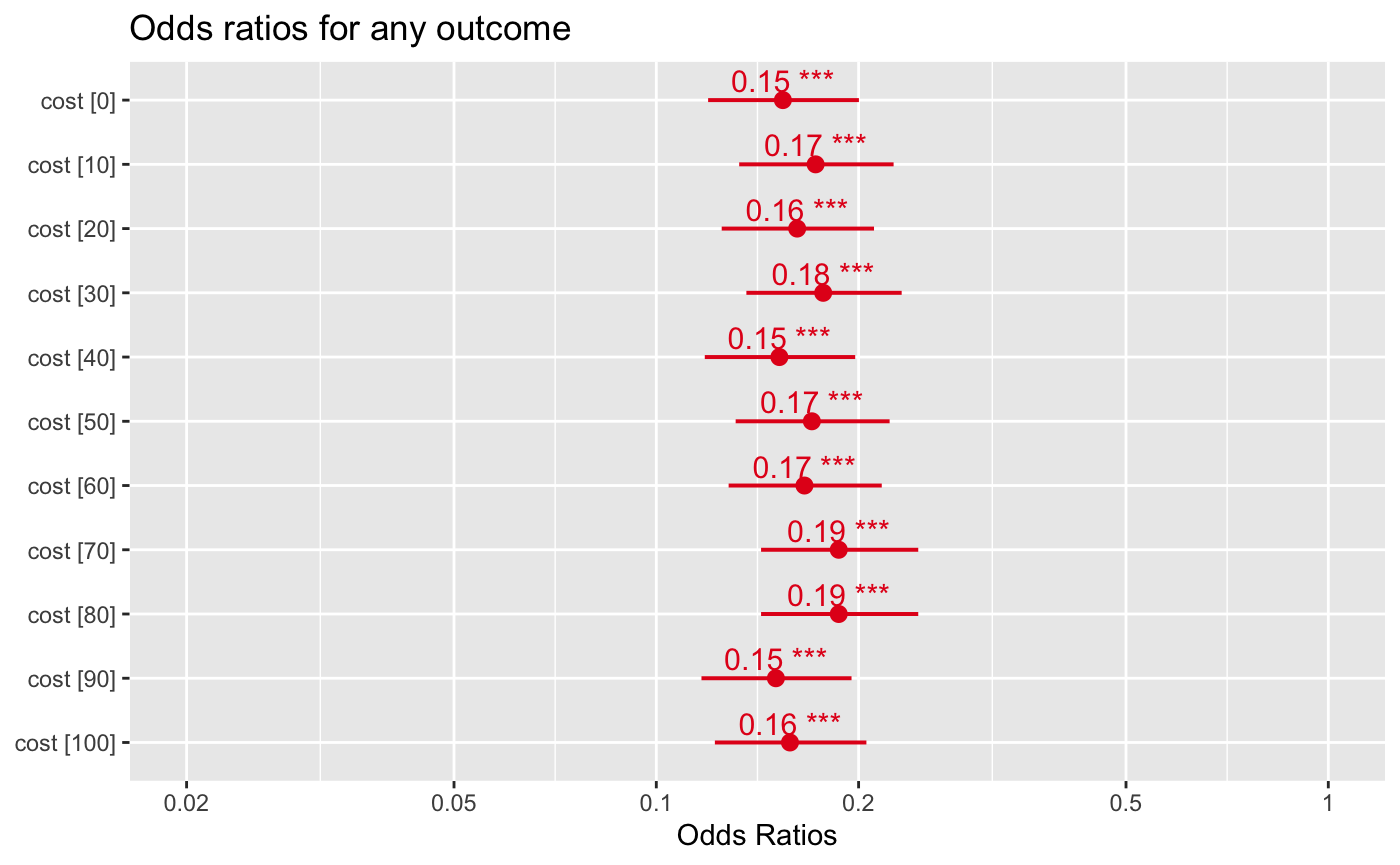


**^S4 Fig. Odds ratios for any trait going into fixation by generation 200, using drift as a reference.^** ^Across the^ *^cost^* ^parameter space, all runs had a much smaller likelihood of seeing any trait in fixation. Parameters: N = 50; traits = 10; generations = 200;^ *^B^* ^= 0.6.^

*Model 2*


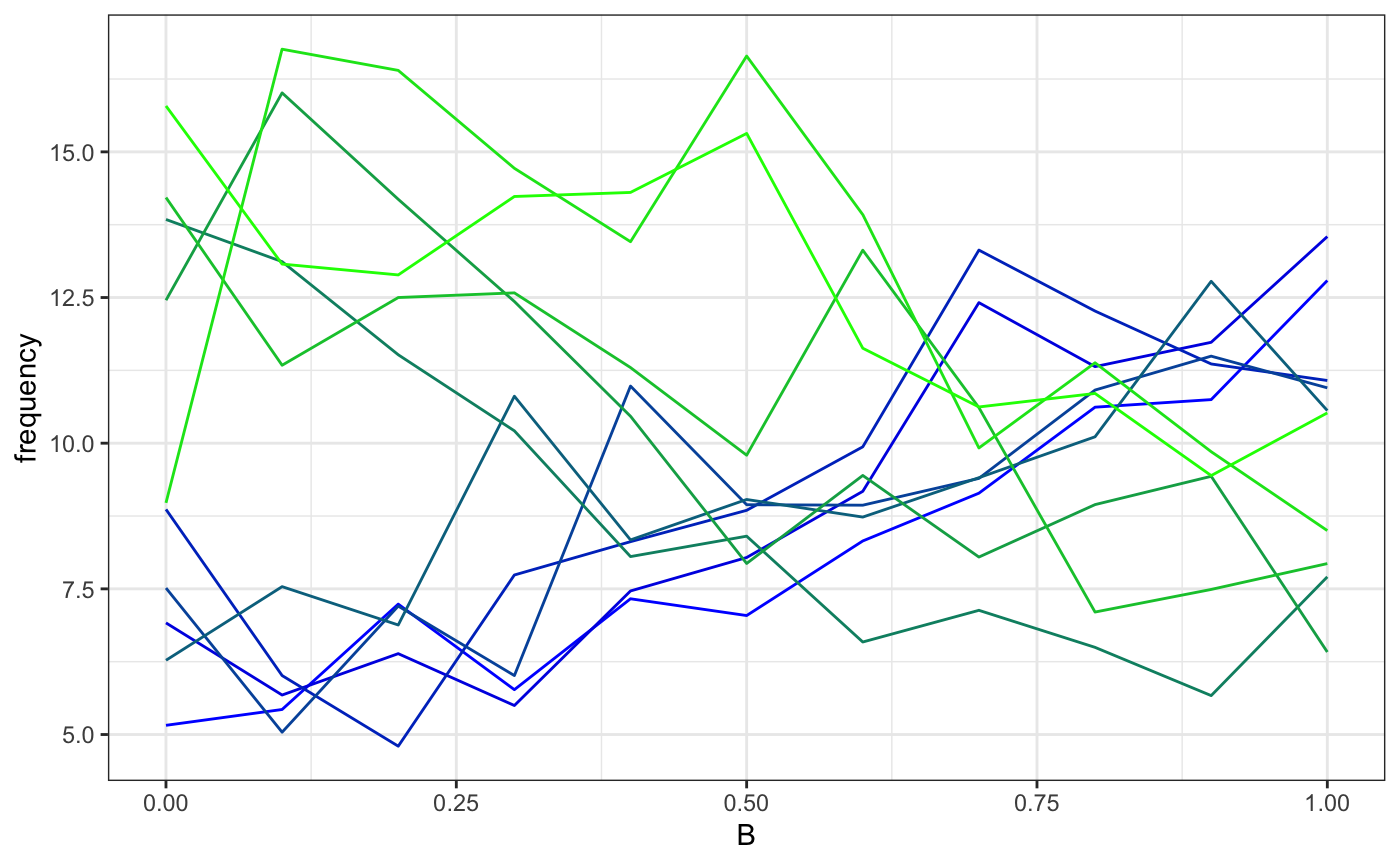


**^S5 Fig. Frequency of green and blue traits in population at generation = 200.^** ^Increasing^ *^B^* ^decreased the frequency of green traits. Parameters: N = 50; traits = 10; generations = 200; cost = 10.^


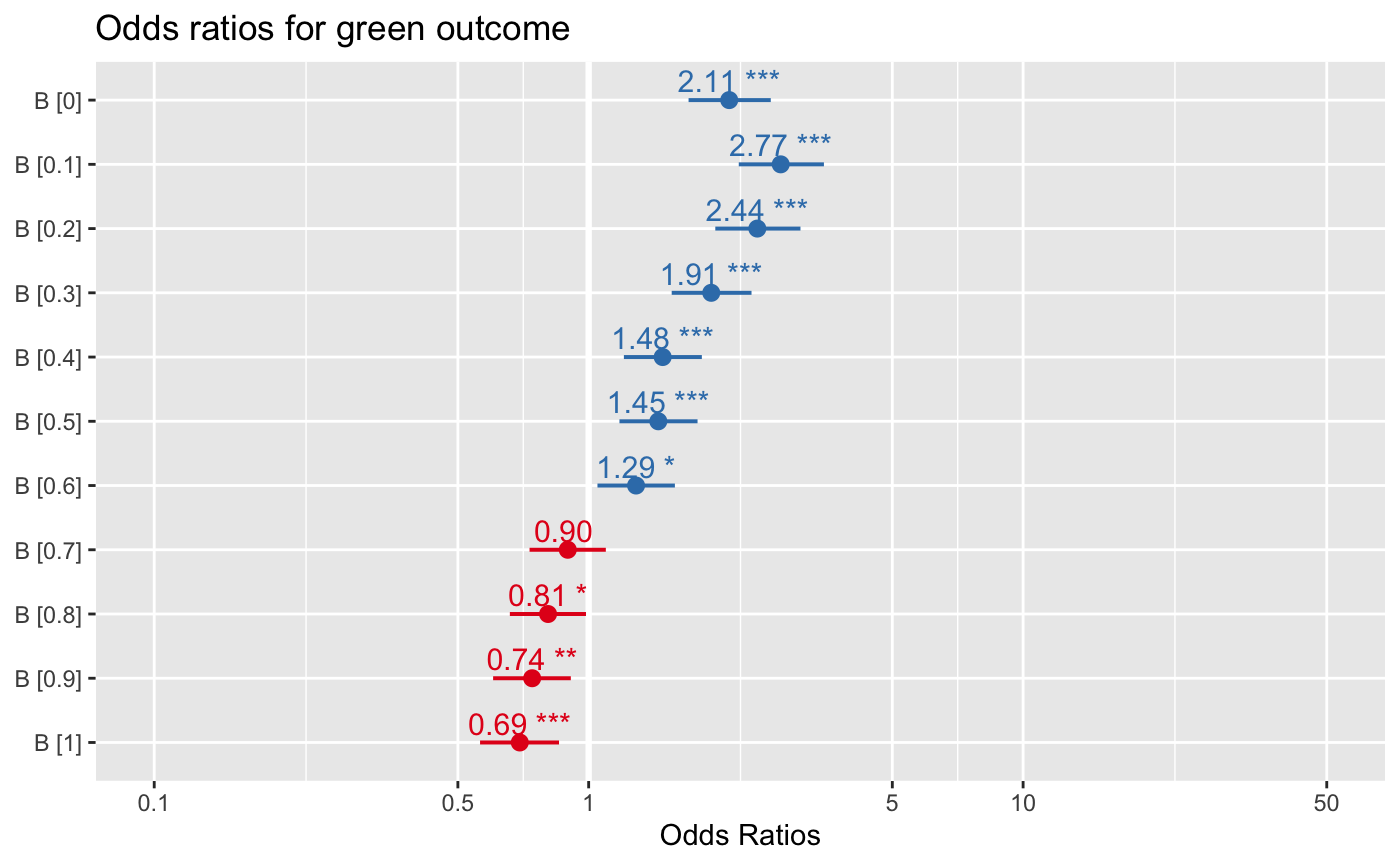


**^S6 Fig. Odds ratios for green vs blue traits going into fixation by generation 200 as^ *^B^* ^increased from 0 to 1.^** ^Parameters: N = 50; traits = 10; generations = 200; cost = 10.^


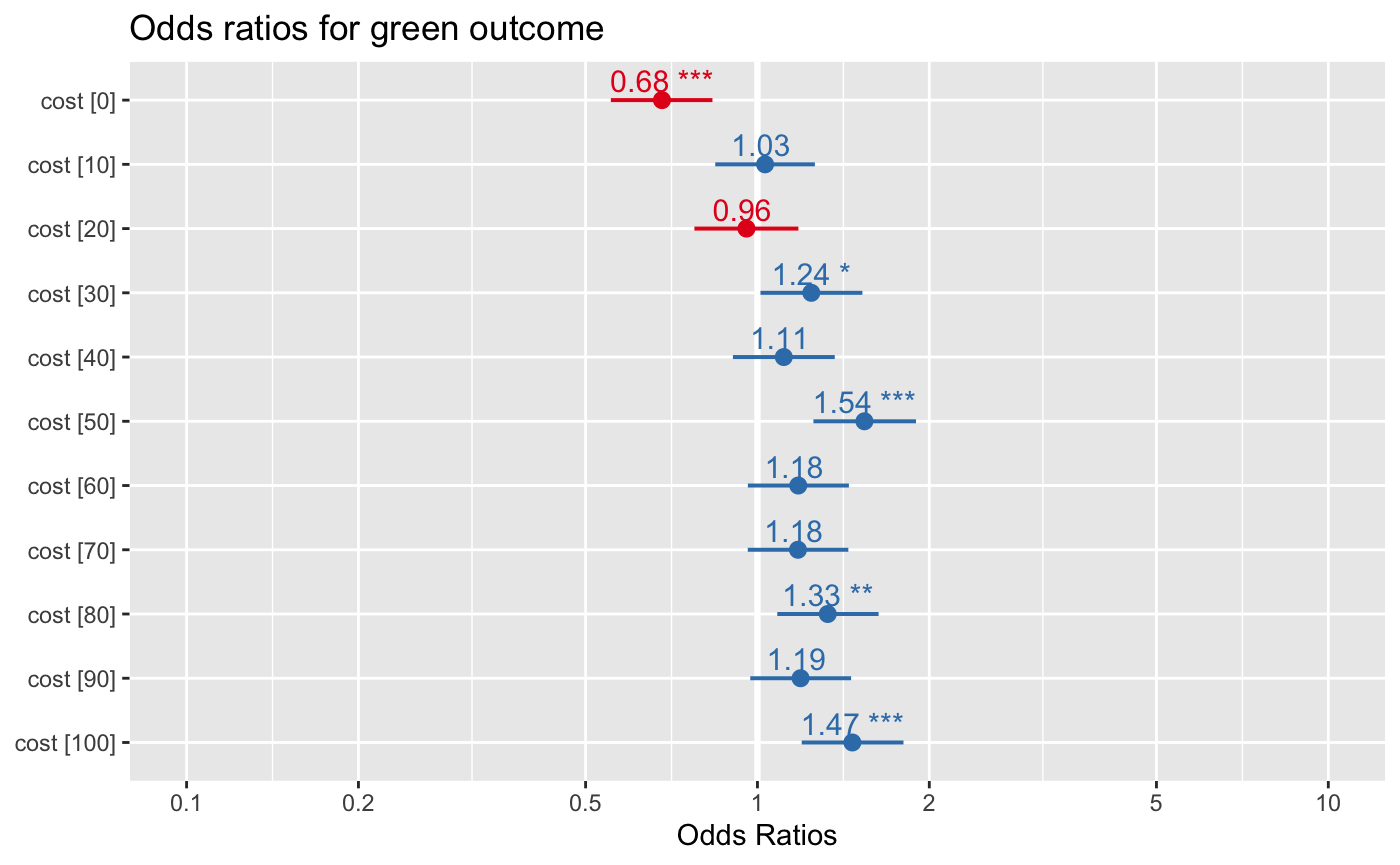


**^S7 Fig. Odds ratios for green vs blue traits going into fixation by generation 200 as cost increased from 0 to 100.^** ^Parameters: N = 50; traits = 10; generations = 200;^ *^B^* ^= 0.7.^

*Model 3*

**^
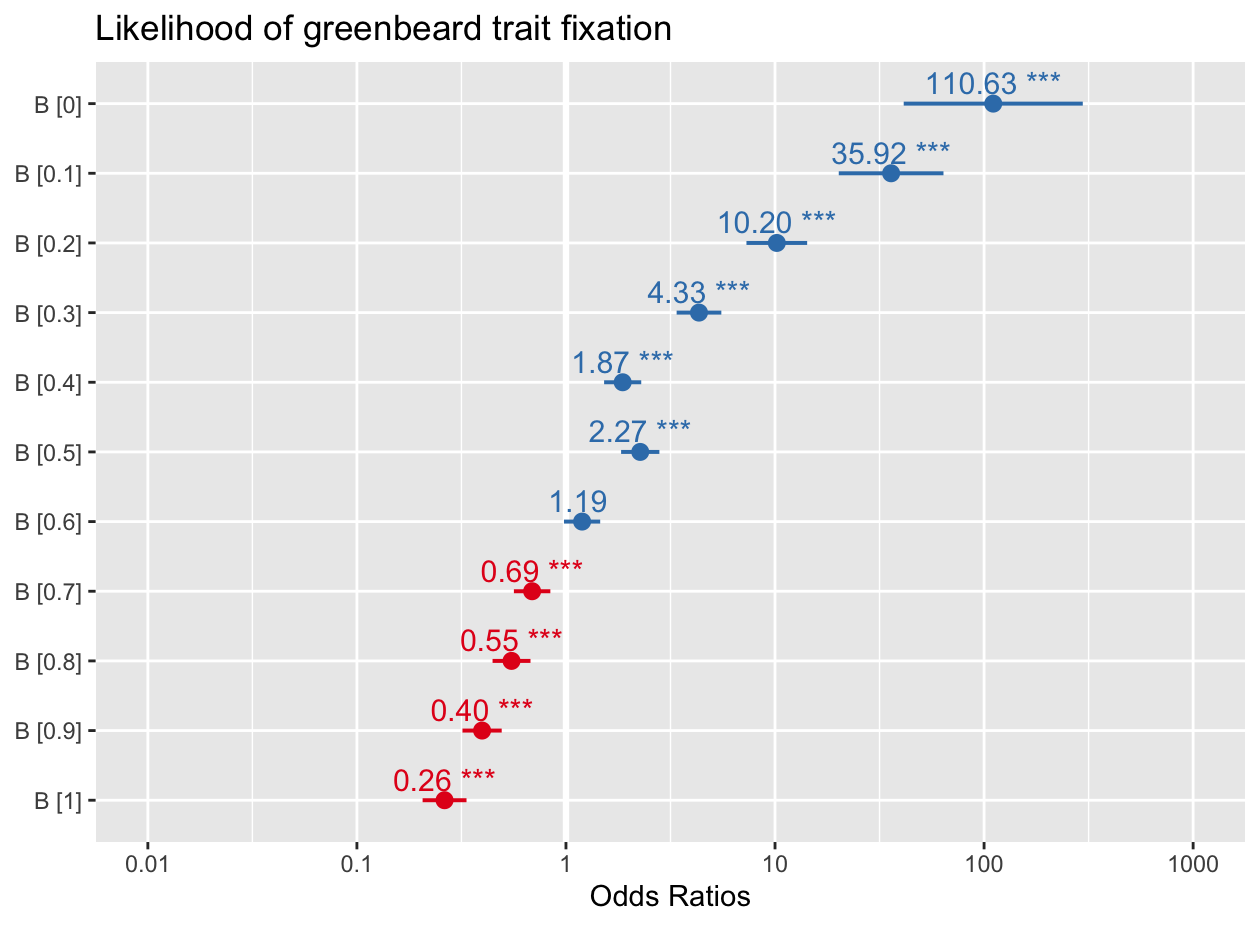
^**

**^S8 Fig. ORs for a green outcome (ie, any green trait going into fixation), compared with drift, as B increases from 0 to 1 in Model 3.^** ^*** indicates P < .001; cost = 10.^


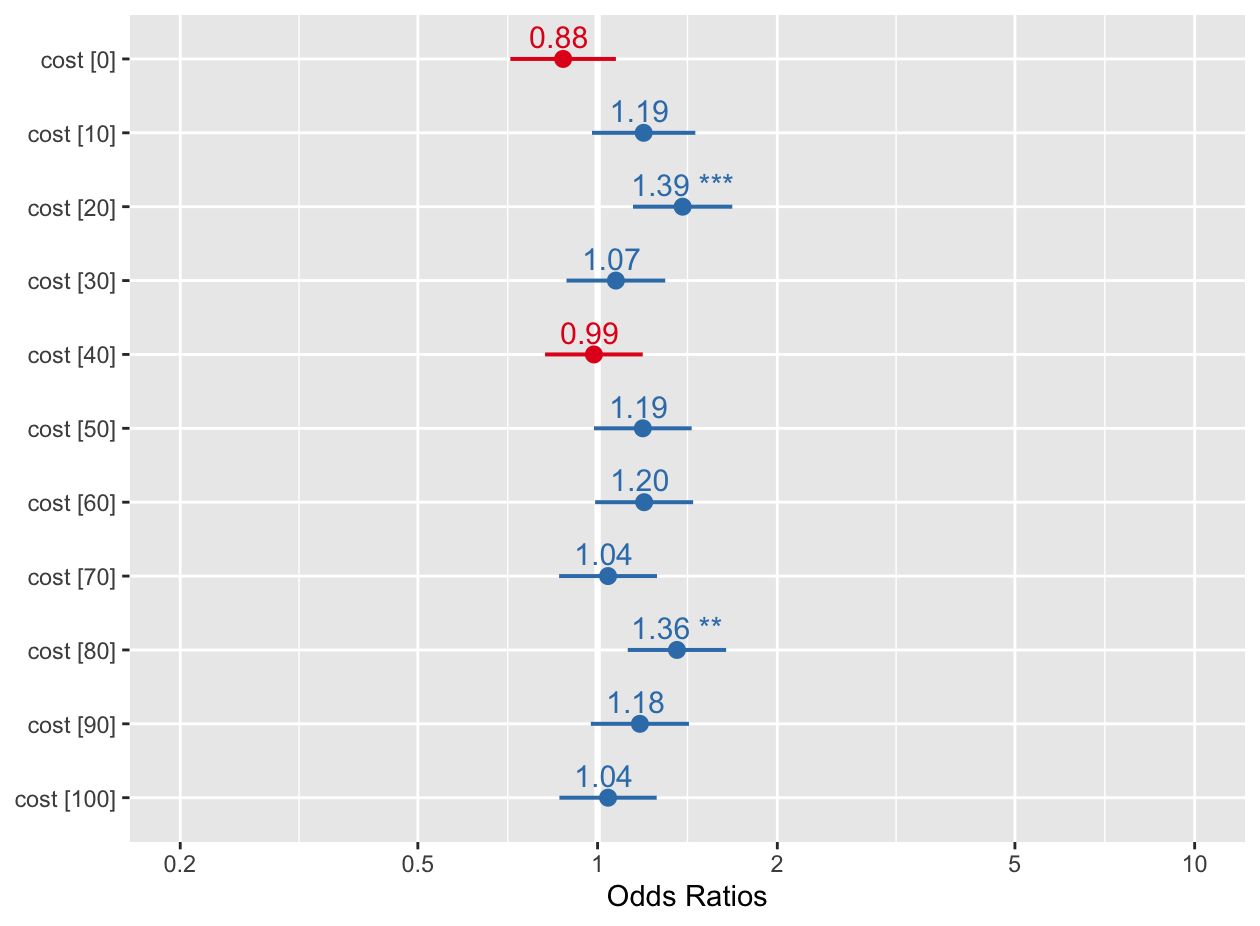


**^S9 Fig. ORs for a green outcome (ie, any green trait going into fixation), compared with drift, as cost increases from 0 to 100 in Model 3.^** ^** indicates P < .01; *** indicates P < .001. Increasing cost did not affect trait distribution where B = 0.6.^

*Model 4*


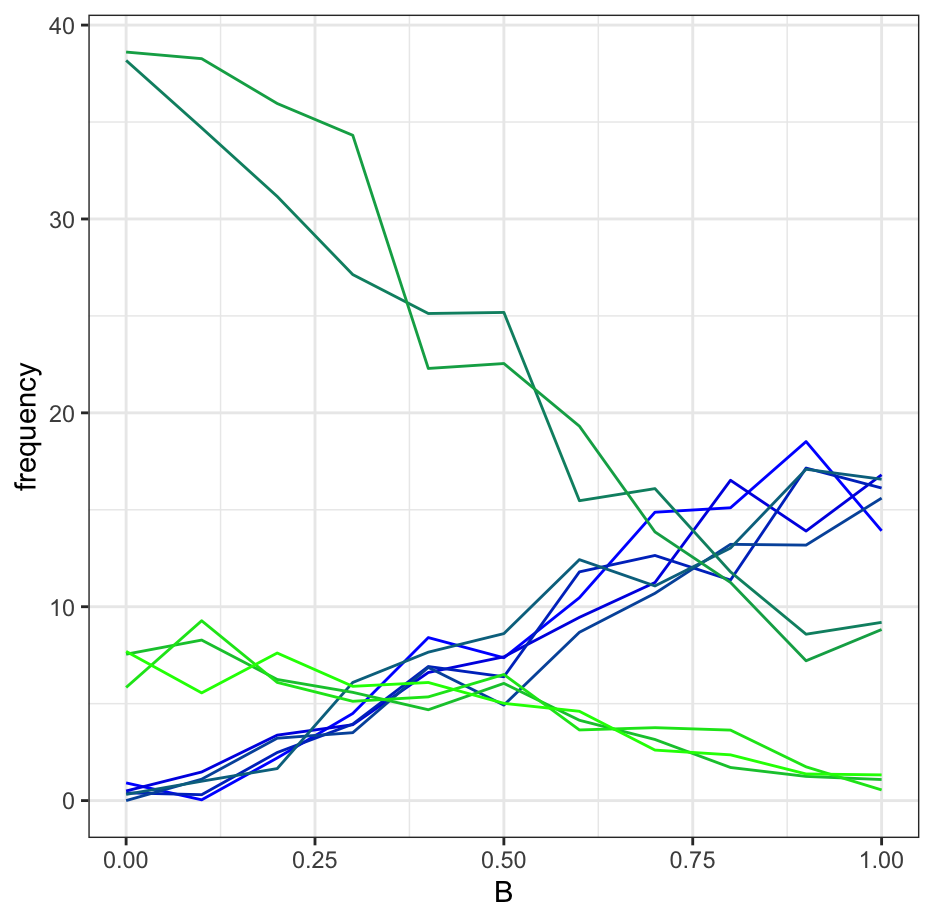


**^S10 Fig. Average trait (of 10 traits on blue-green spectrum) frequency in Model 4.^** ^x-axis = value of^ *^B^*^, increasing from 0 to 1 in increments of 0.1; 500 runs per value; y-axis = average frequency of each trait at final generation in a population of 100 individuals. The frequency of individuals with a green trait at final generation drops with each 0.1 increase of^ *^B^*^. We held^ *^cost^* ^steady at 10.^

*Model 5*


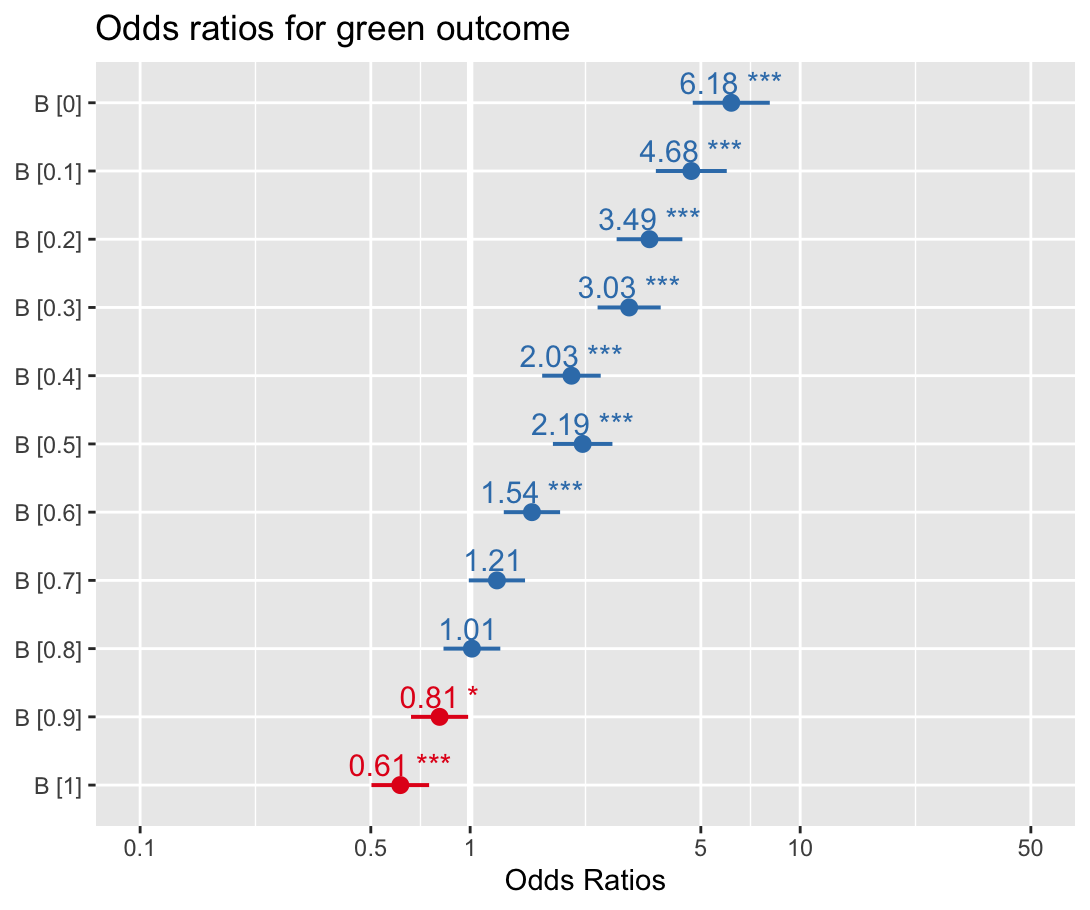


**^S11 Fig. ORs for a green outcome, compared with drift, as B increases from 0 to 1 in Model 5.^** ^* indicates^ *^P^* ^< .05; *** indicates^ *^P^* ^< .001. The^ *^B^* ^variable is a strong predictor of which trait-group goes into fixation; the traits are, again, but only distributed non-significantly when^ *^B^* ^= 0.7 or 0.8.^

**^
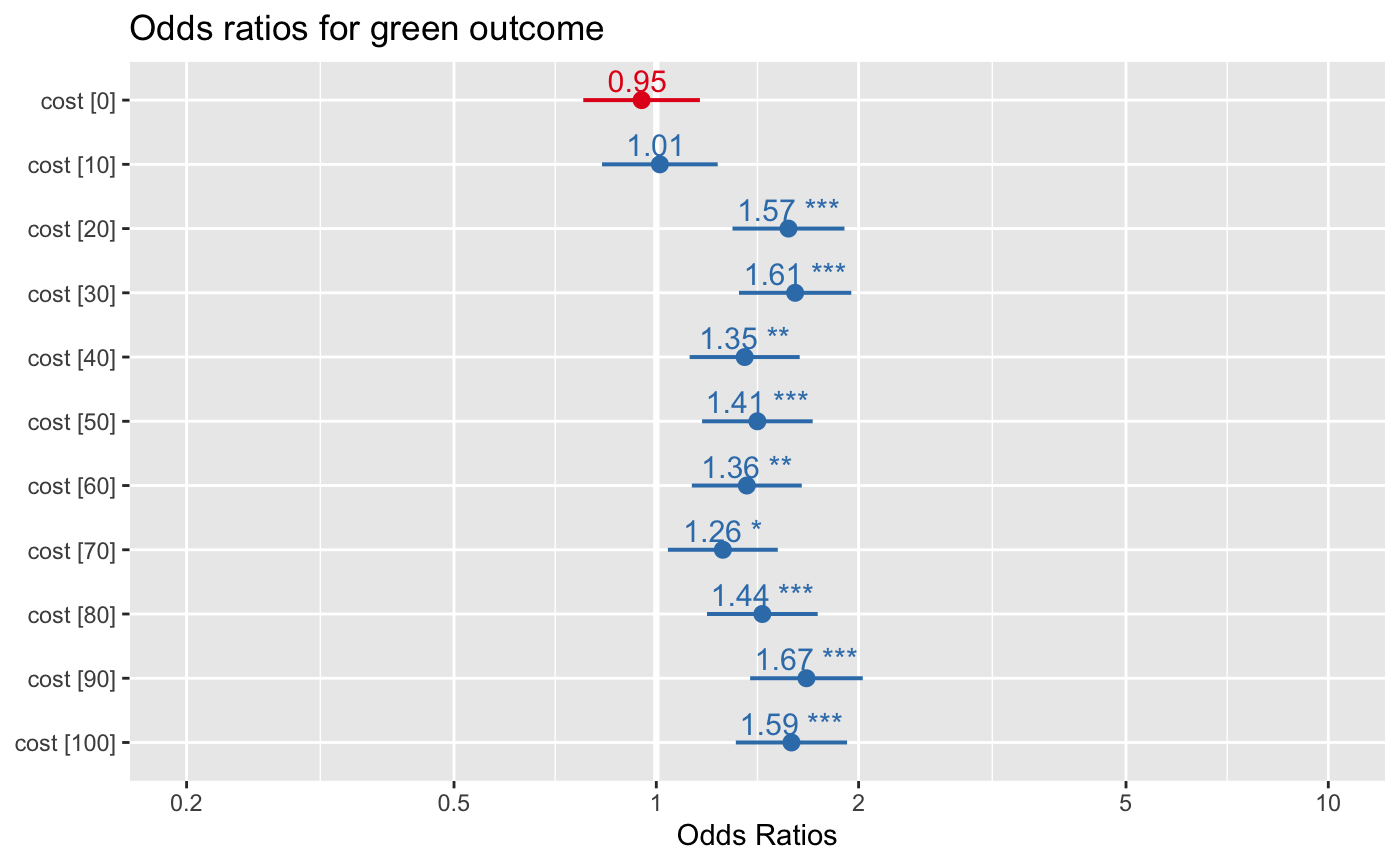
^**

**^S12 Fig. ORs for a green outcome, compared with drift, as^ *^cost^* ^increases from 0 to 100 in Model 5.^** ^* indicates^ *^P^* ^< .05; ** indicates^ *^P^* ^< .01; *** indicates^ *^P^* ^< .001. Varying^ *^cost^* ^did not affect likelihood of a green outcome.^


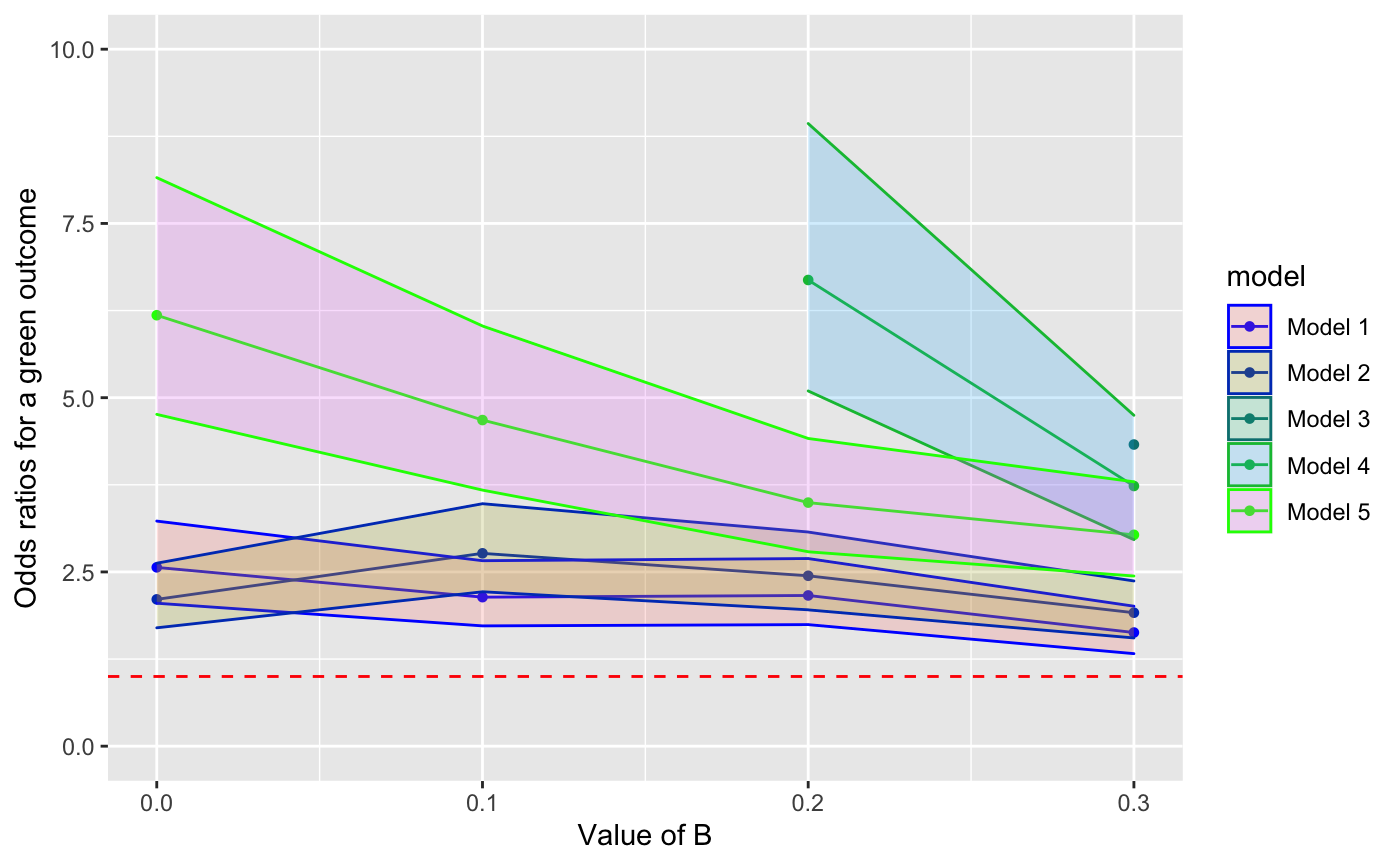


**^S13 Fig. ORs, across all 5 models, for a green outcome (y-axis) as B increases from 0 to 0.3 (zoomed in for viewability); shaded areas represent 95% CIs.^**

**^
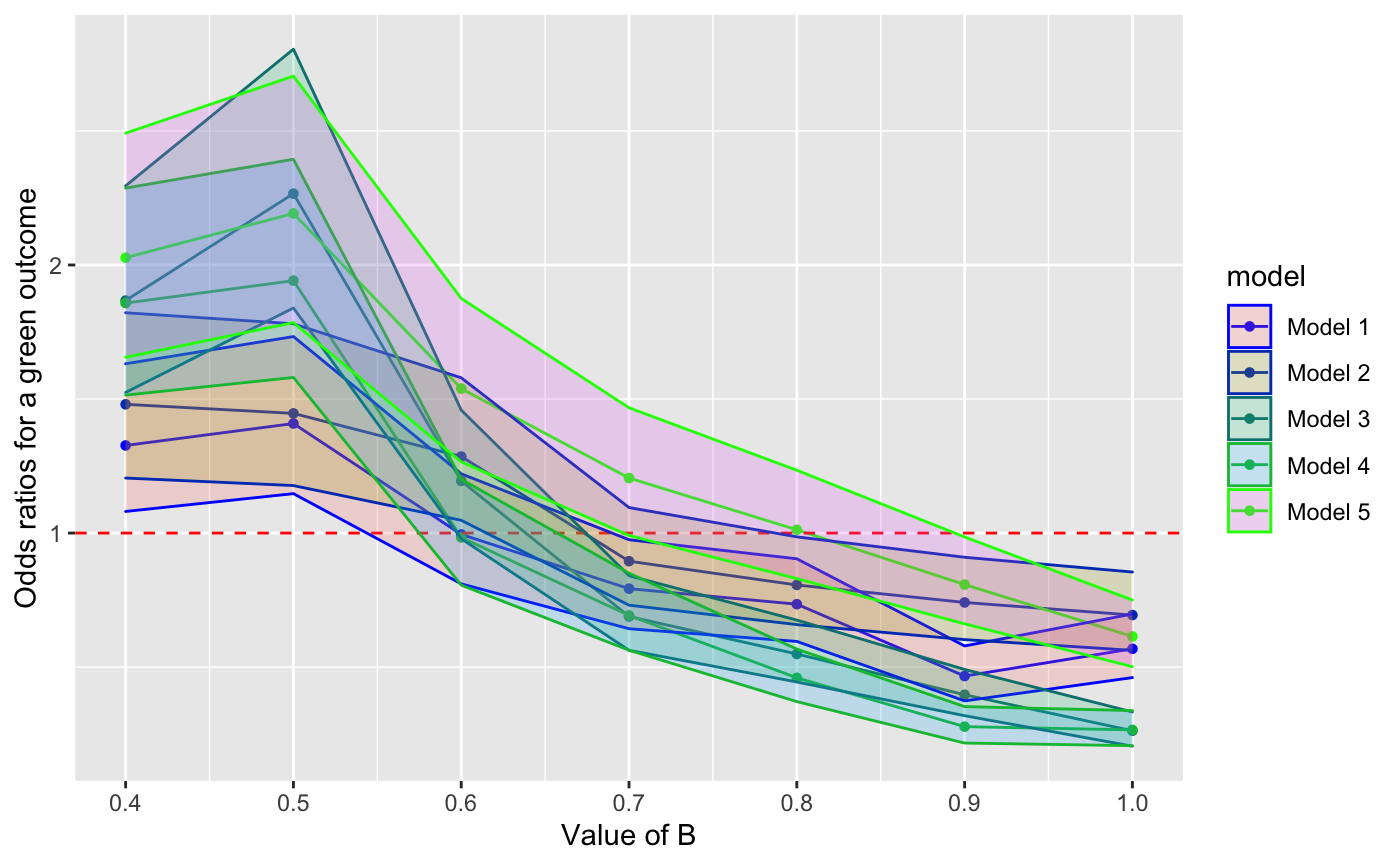
^**

**^S14 Fig. ORs, across all 5 models, for a green outcome (y-axis) as B increases from 0 to 1; shaded areas represent 95% CIs.^**
