# Supplementary figures and images for: Shibboleth: An agent-based model of signalling mimicry
Source: PLoS One. 2023 Jul 31;18(7):e0289333. doi: 10.1371/journal.pone.0289333 (PMC10389733; doi:10.1371/journal.pone.0289333)

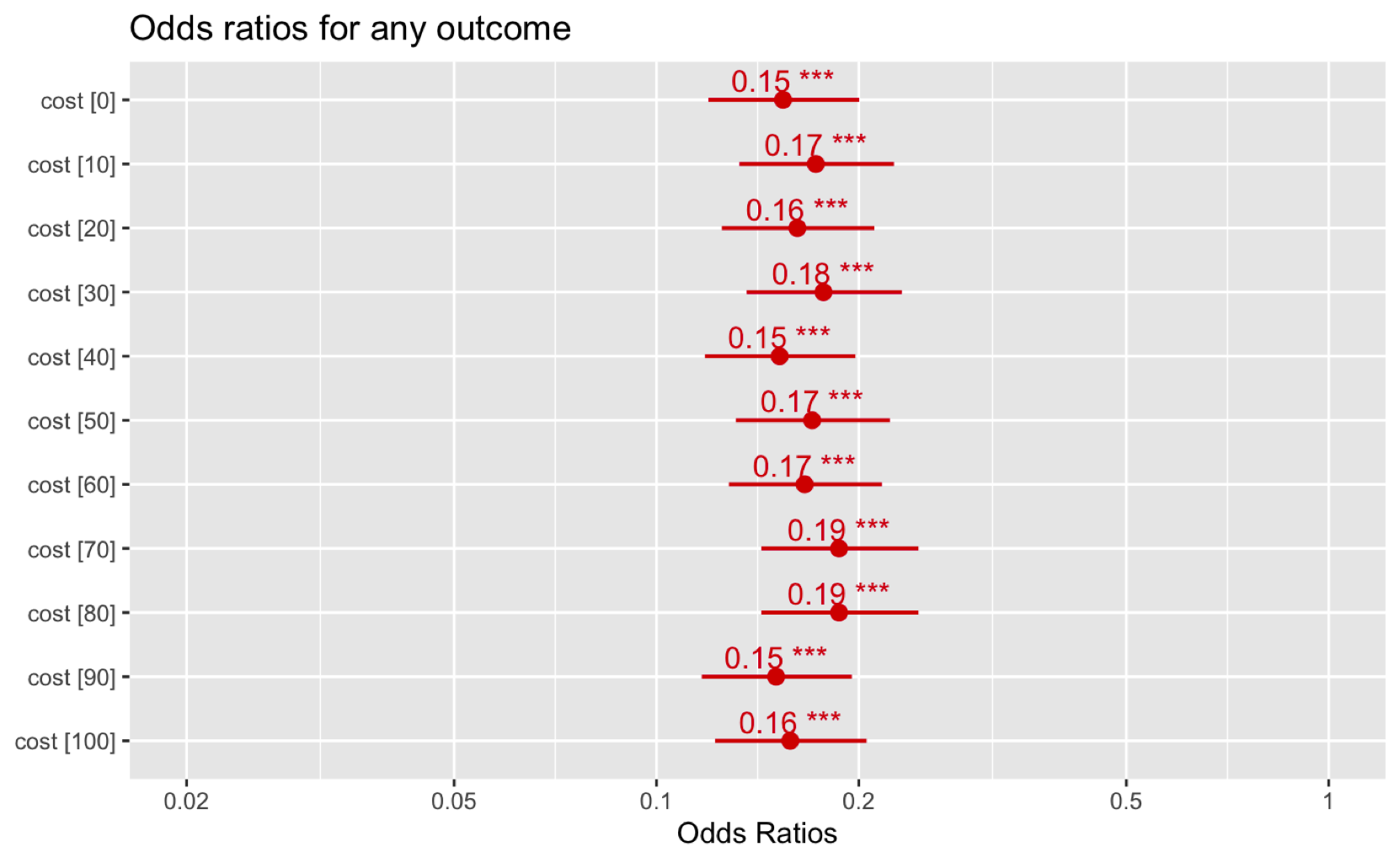

Supplement: S1 File — (ZIP) [file pone.0289333.s001.zip › Supporting-information/S4_Fig.tif]

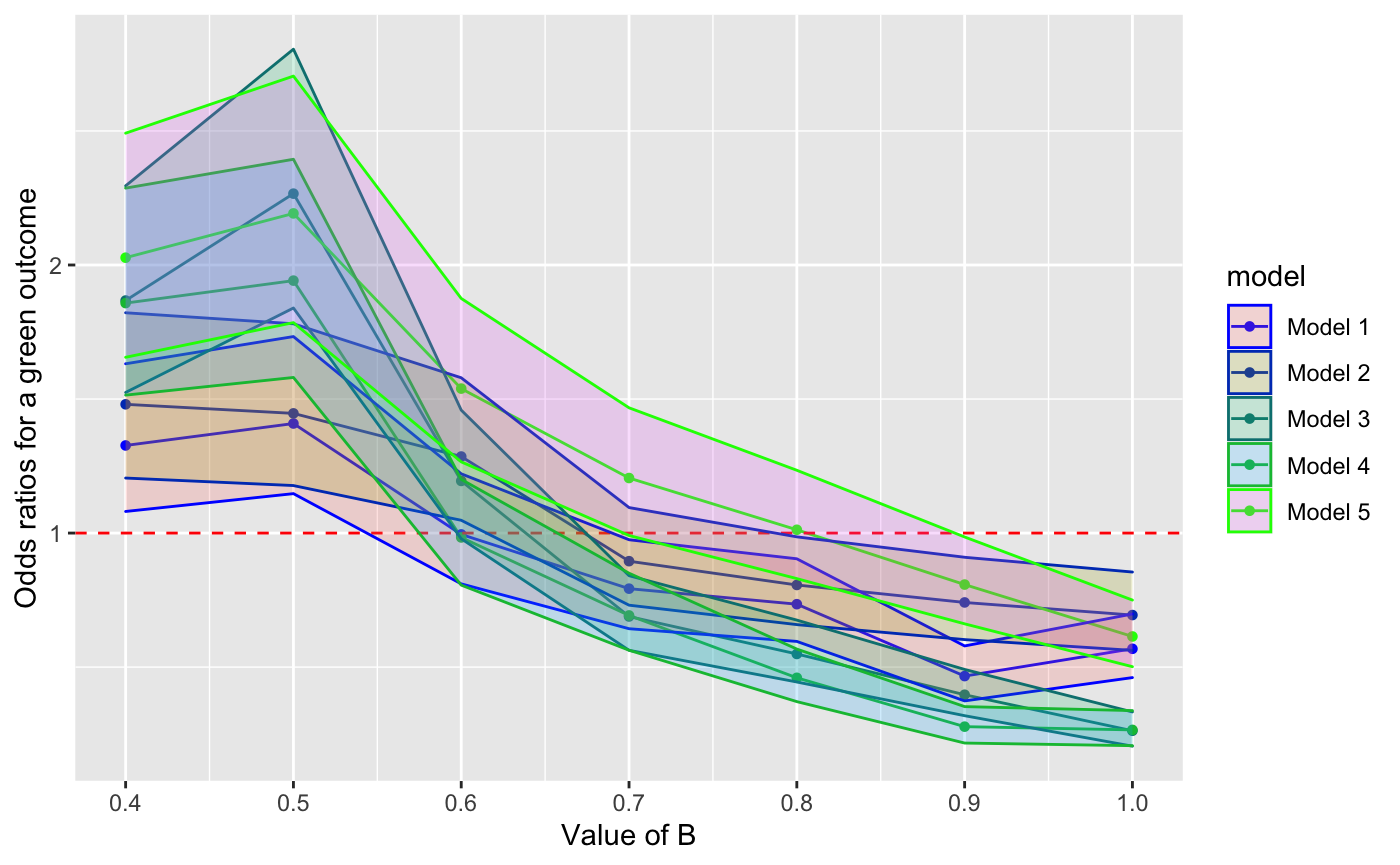

Supplement: S1 File — (ZIP) [file pone.0289333.s001.zip › Supporting-information/S14_Fig.tif]

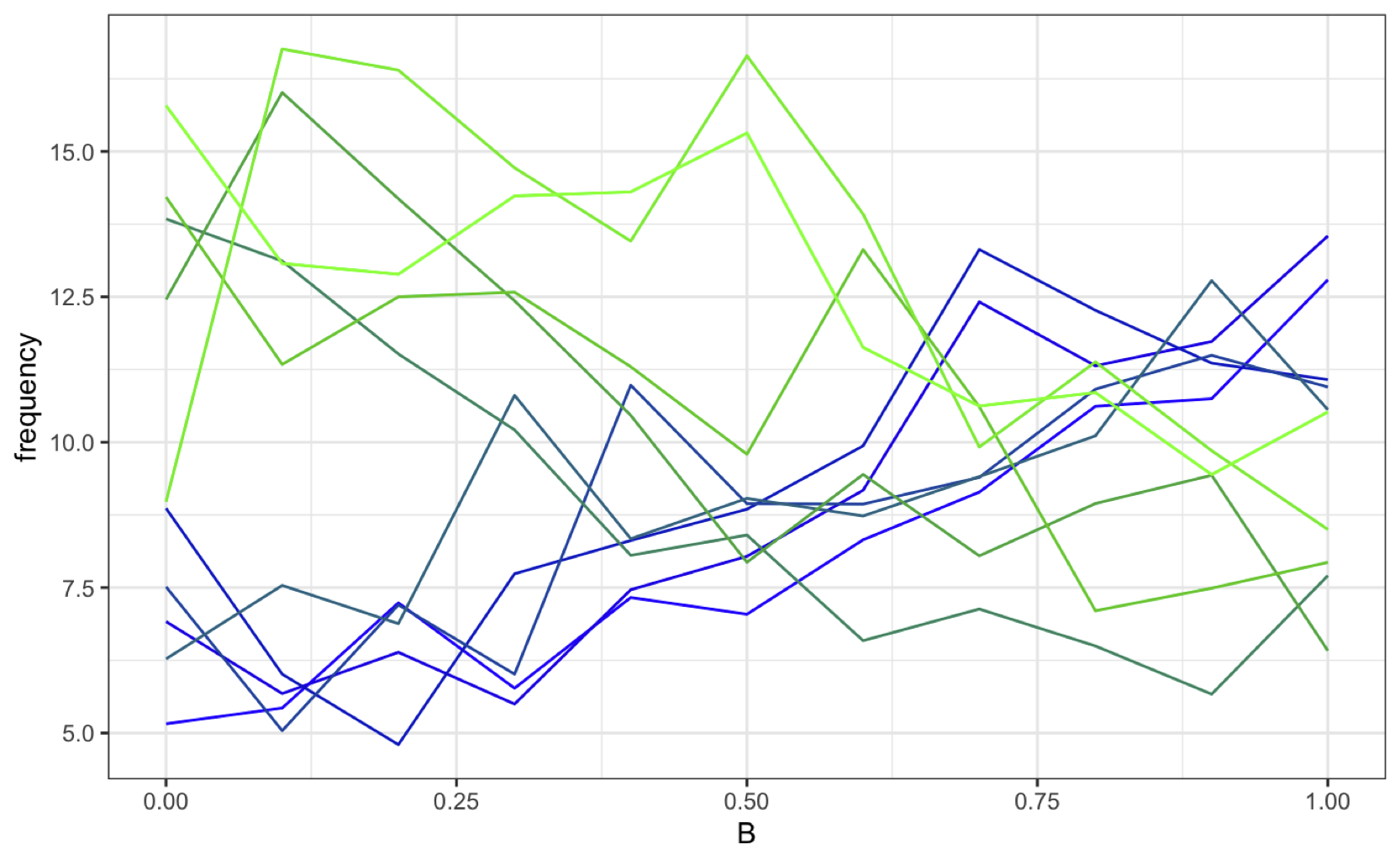

Supplement: S1 File — (ZIP) [file pone.0289333.s001.zip › Supporting-information/S5_Fig.tif]

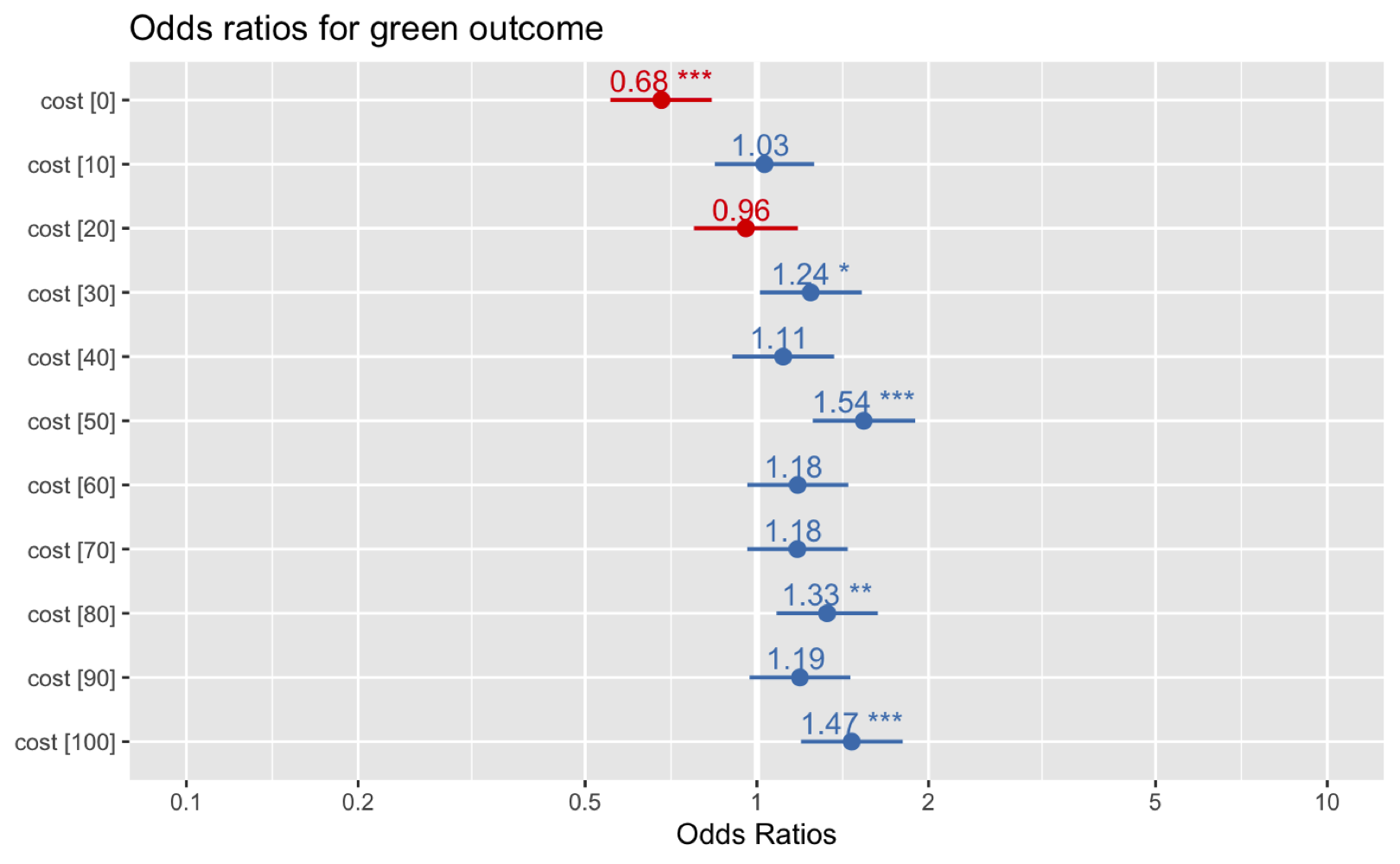

Supplement: S1 File — (ZIP) [file pone.0289333.s001.zip › Supporting-information/S7_Fig.tif]

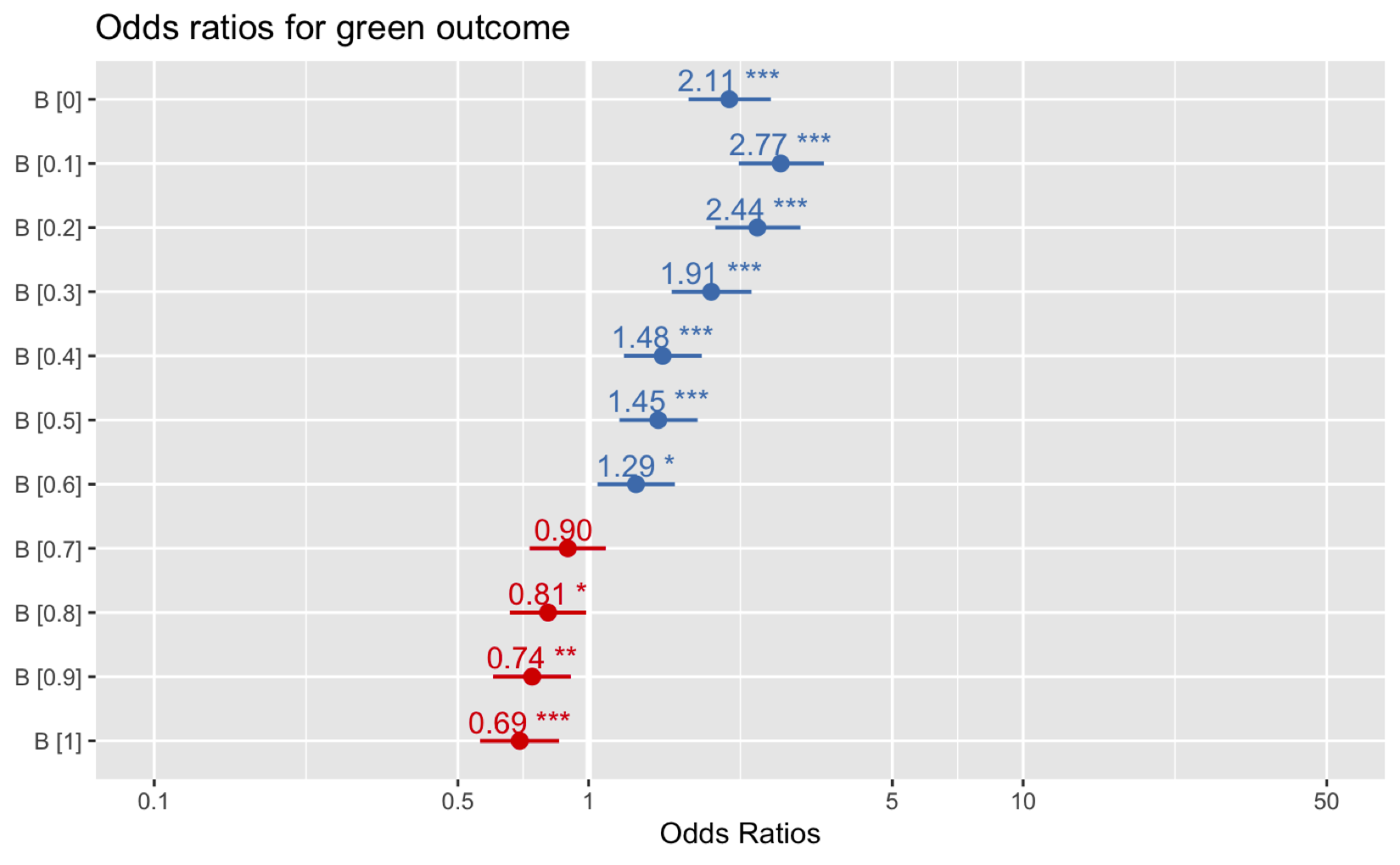

Supplement: S1 File — (ZIP) [file pone.0289333.s001.zip › Supporting-information/S6_Fig.tif]

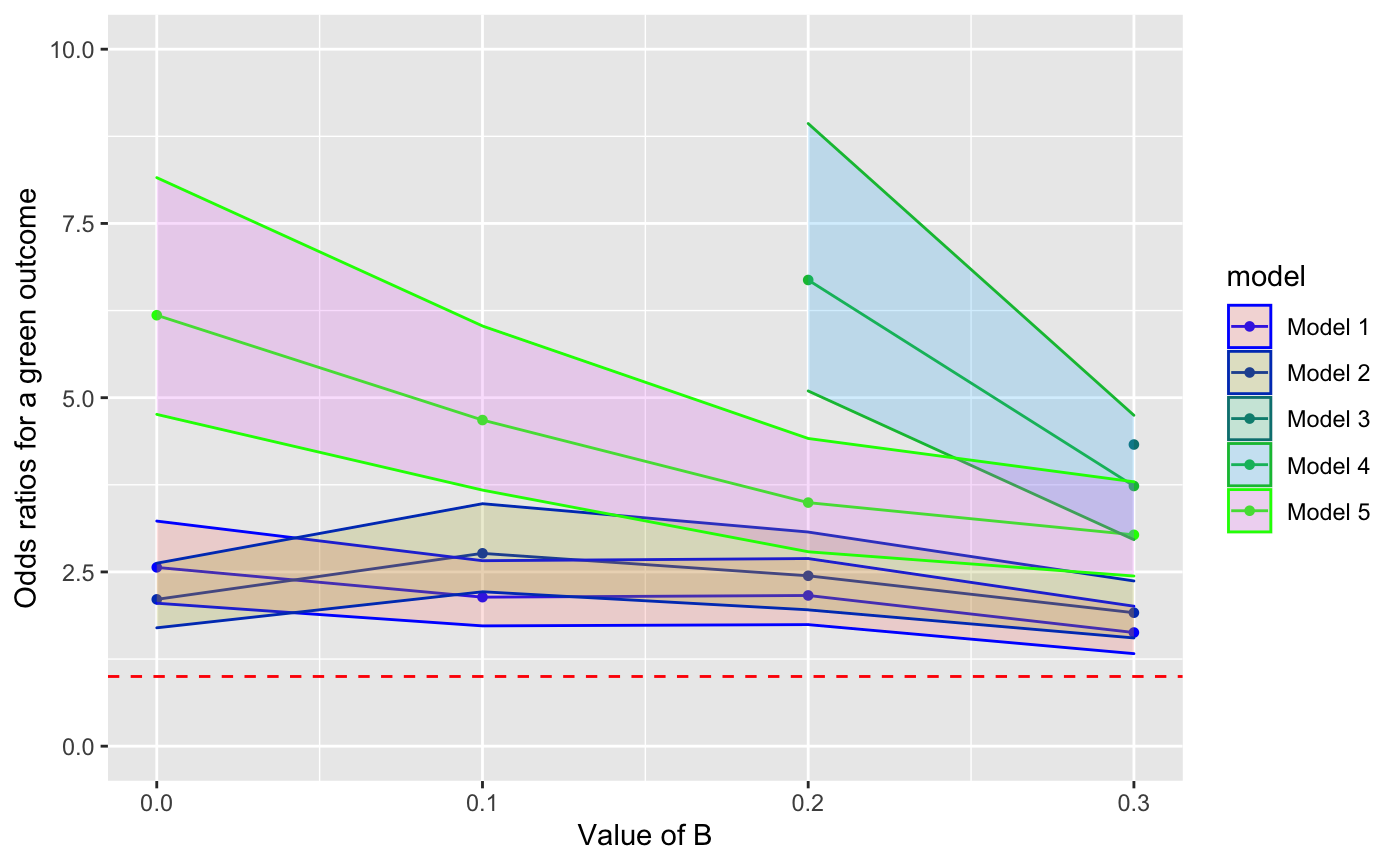

Supplement: S1 File — (ZIP) [file pone.0289333.s001.zip › Supporting-information/S13_Fig.tif]

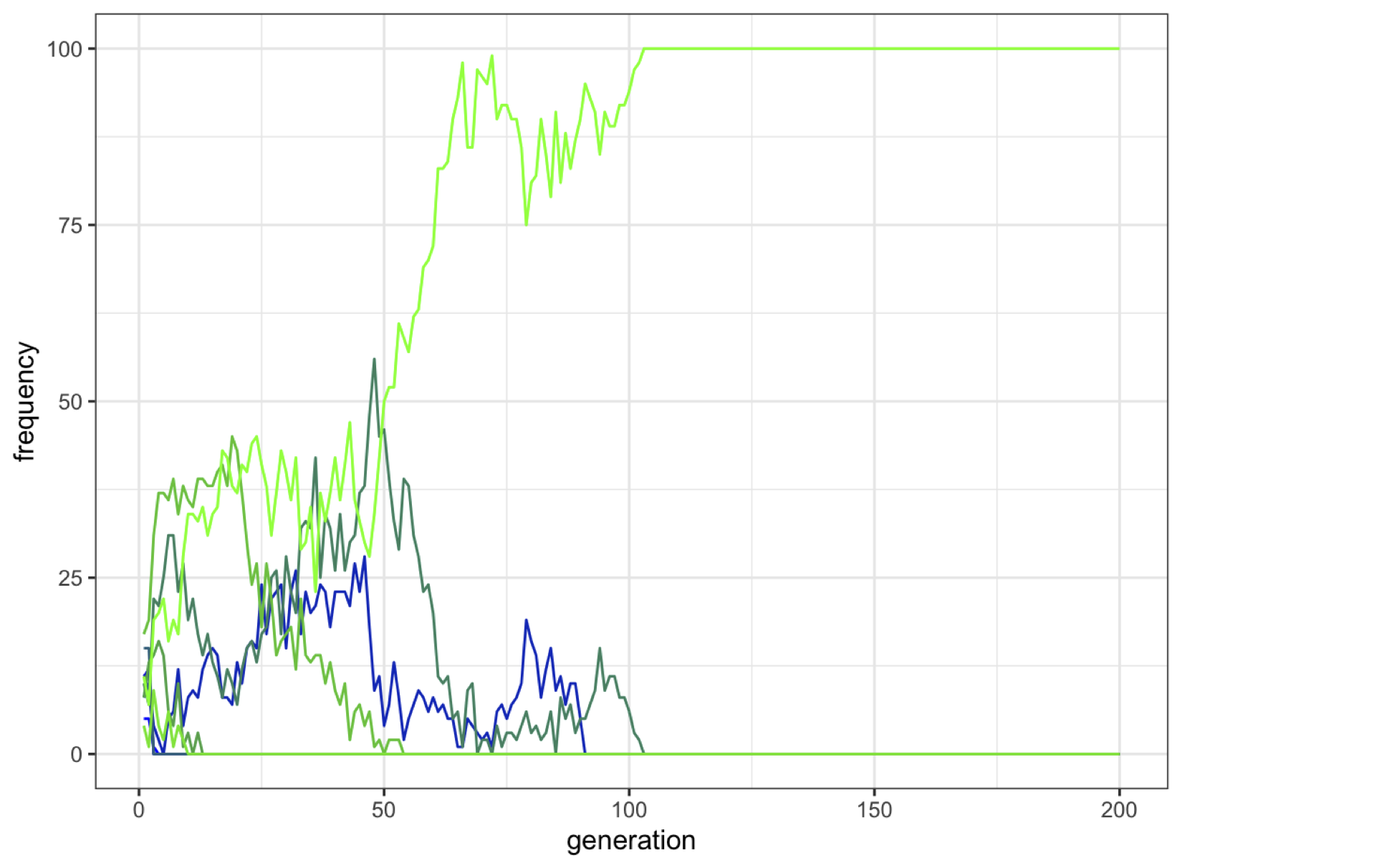

Supplement: S1 File — (ZIP) [file pone.0289333.s001.zip › Supporting-information/S3_Fig.tif]

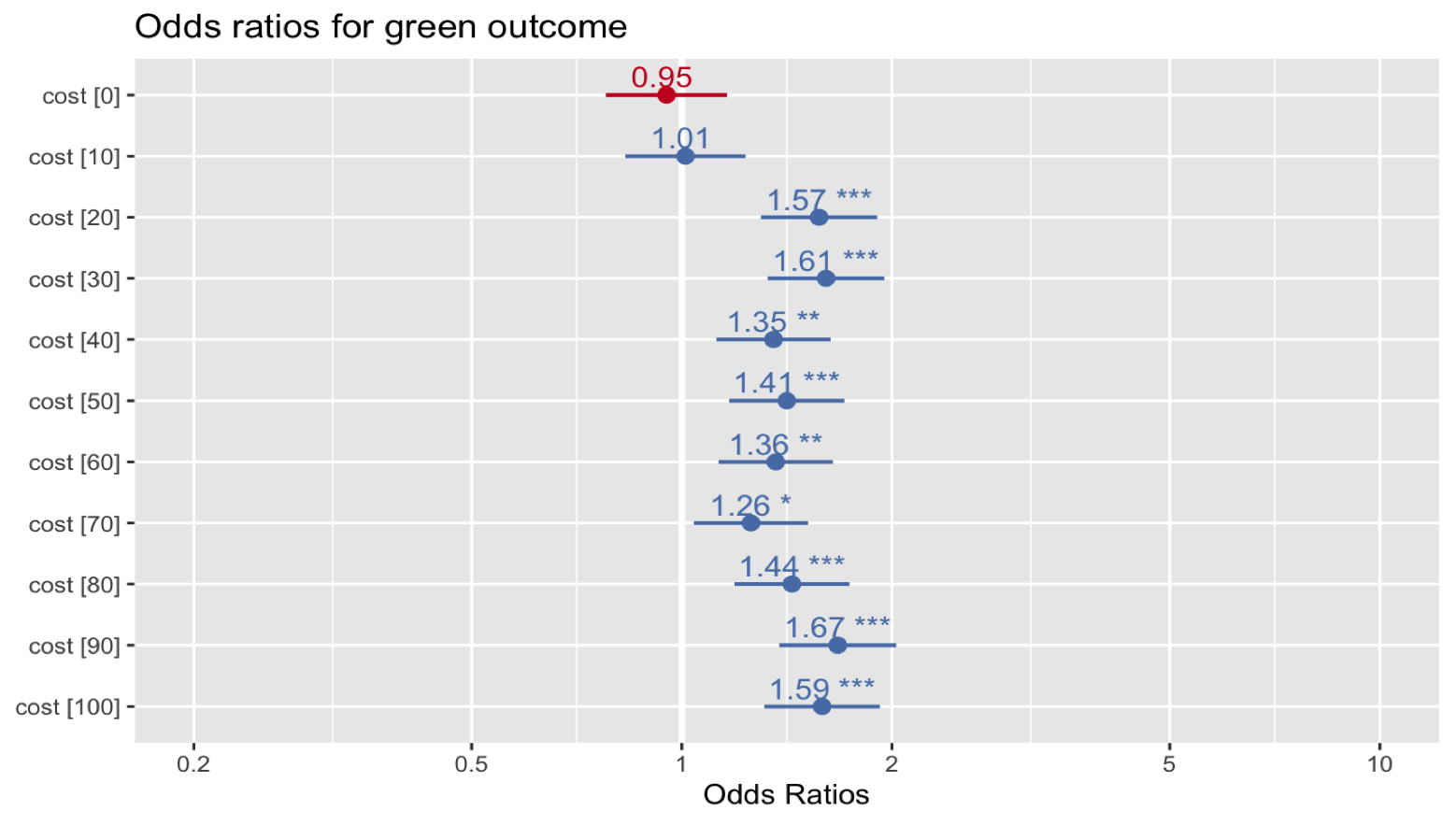

Supplement: S1 File — (ZIP) [file pone.0289333.s001.zip › Supporting-information/S12_Fig.tif]

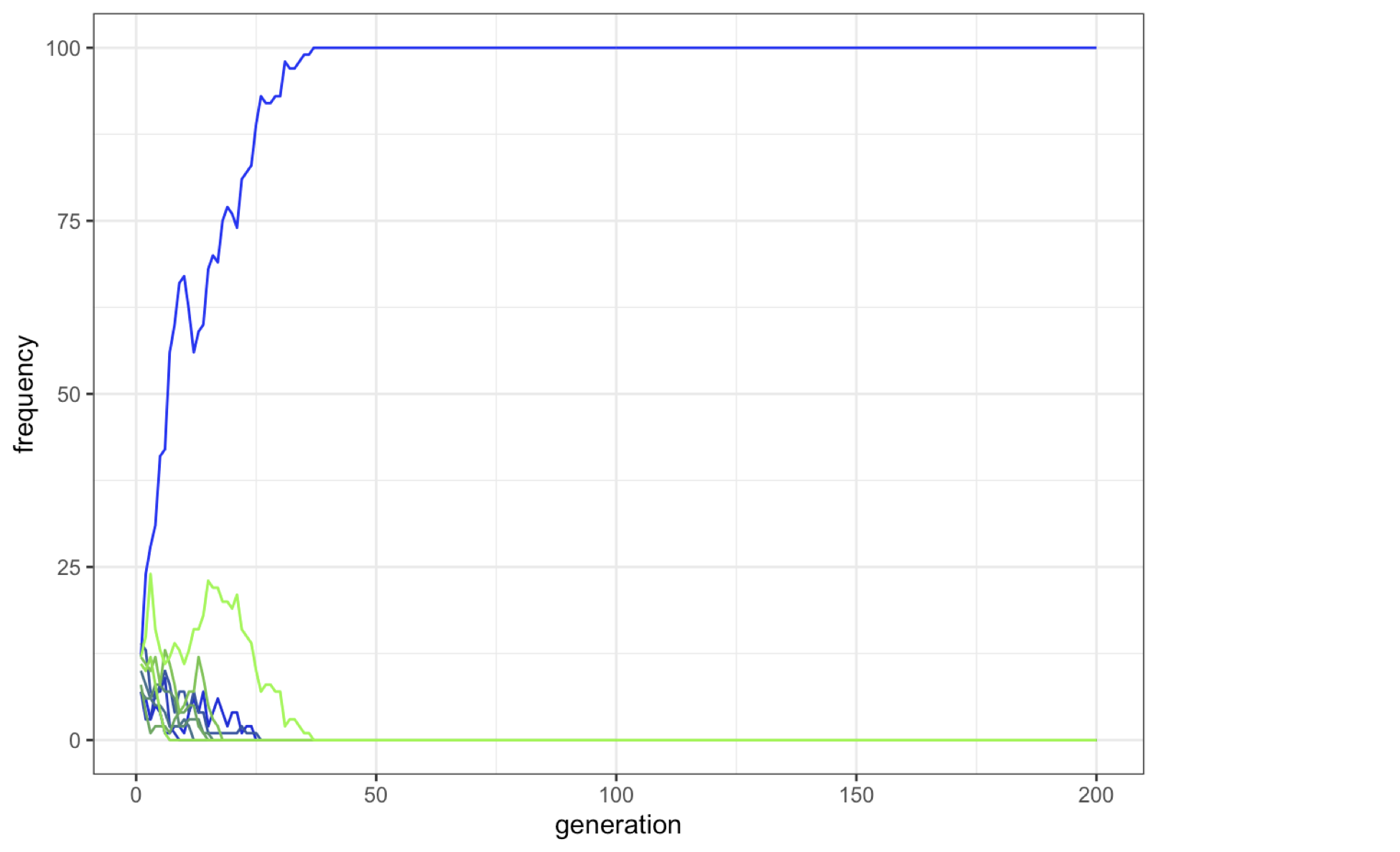

Supplement: S1 File — (ZIP) [file pone.0289333.s001.zip › Supporting-information/S2_Fig.tif]

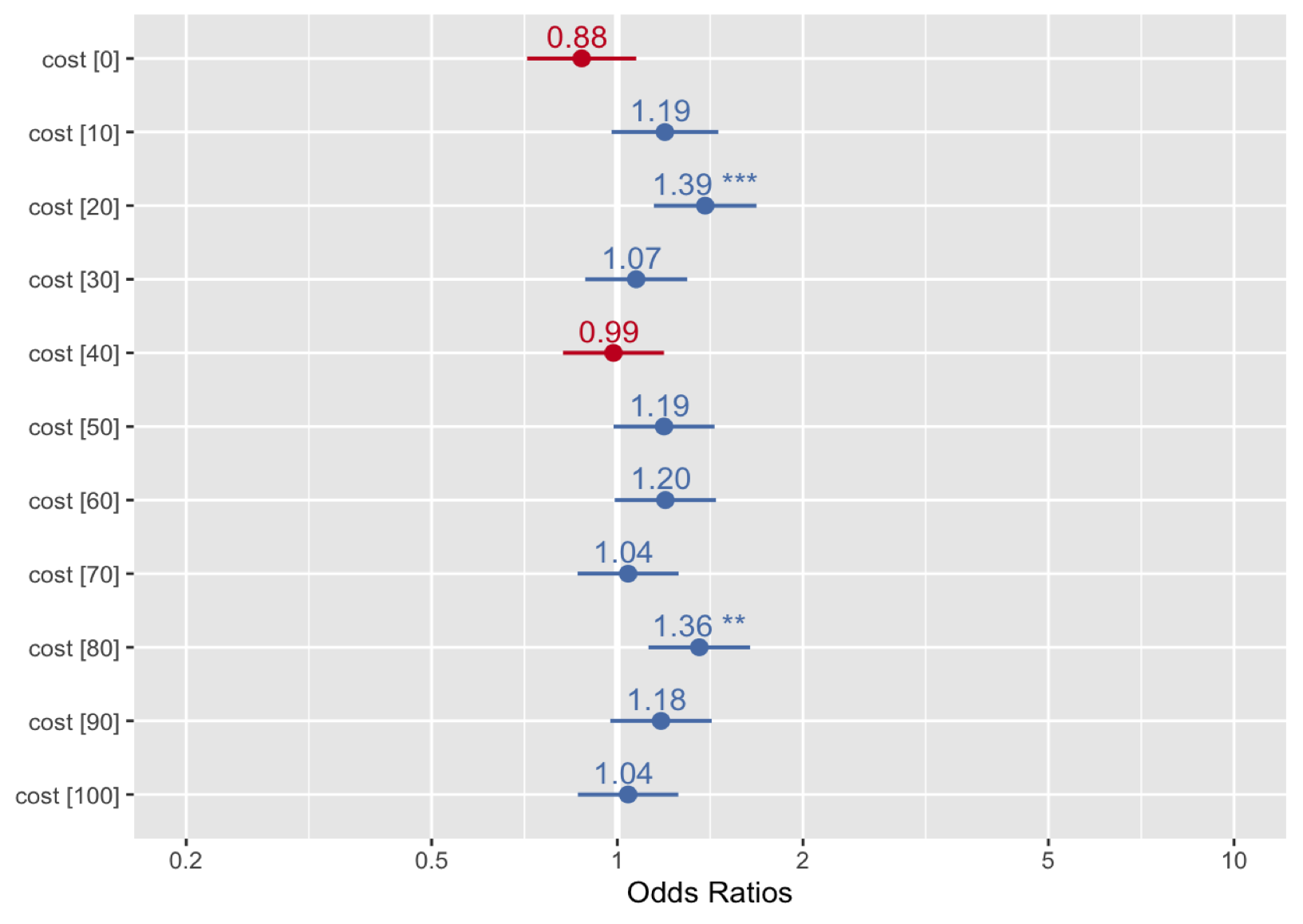

Supplement: S1 File — (ZIP) [file pone.0289333.s001.zip › Supporting-information/S9_Fig.tif]

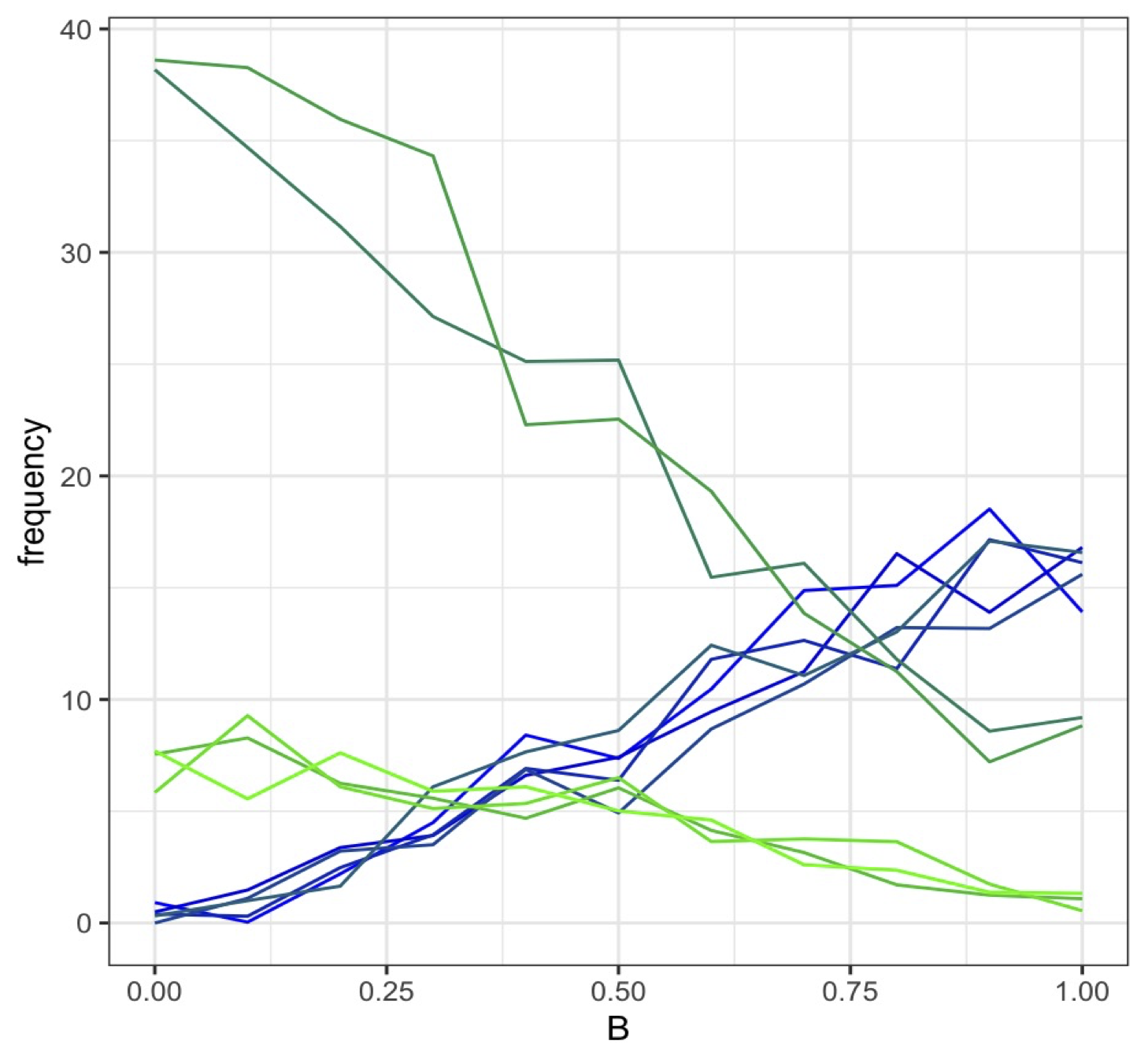

Supplement: S1 File — (ZIP) [file pone.0289333.s001.zip › Supporting-information/S10_Fig.tif]

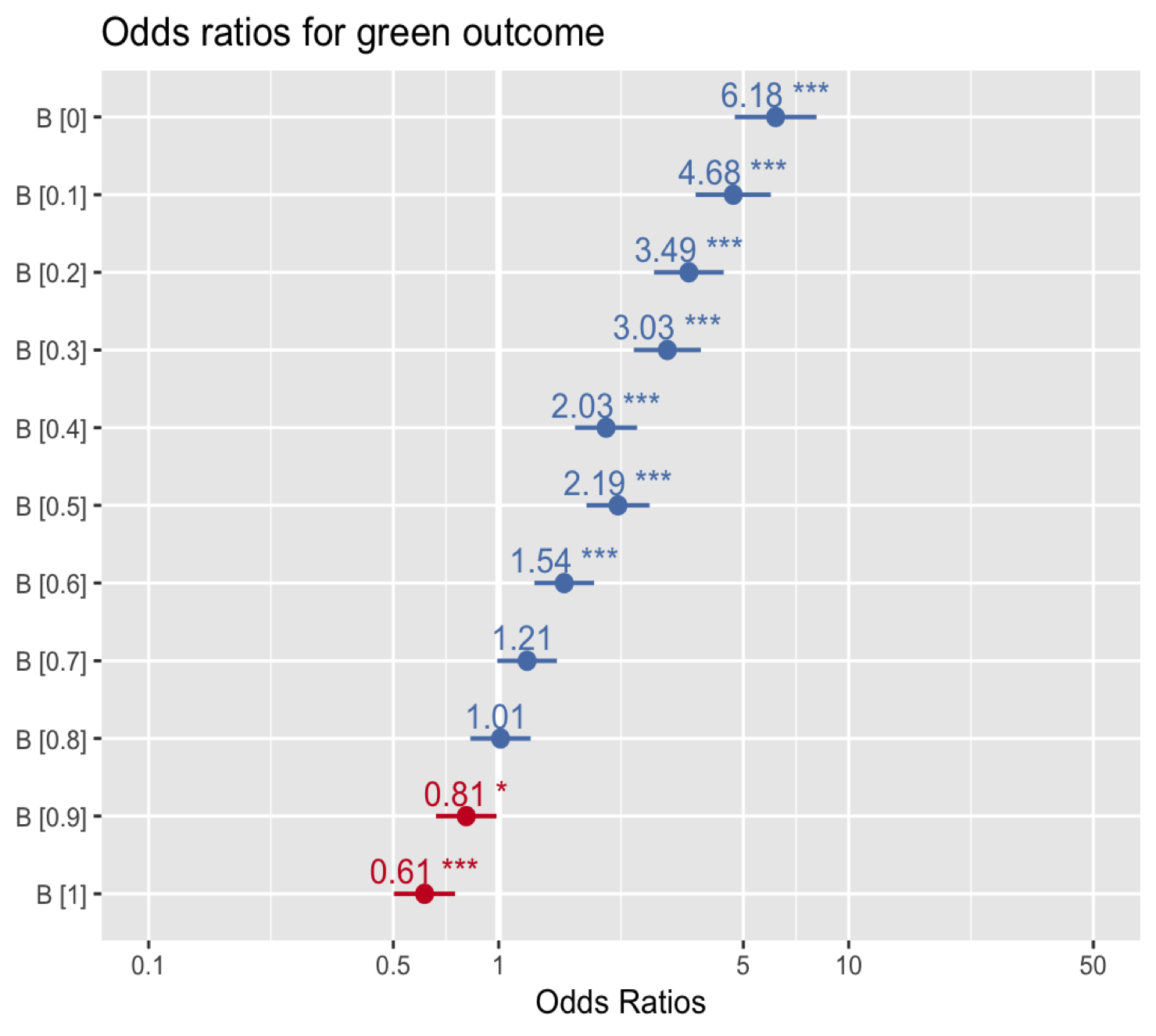

Supplement: S1 File — (ZIP) [file pone.0289333.s001.zip › Supporting-information/S11_Fig.tif]

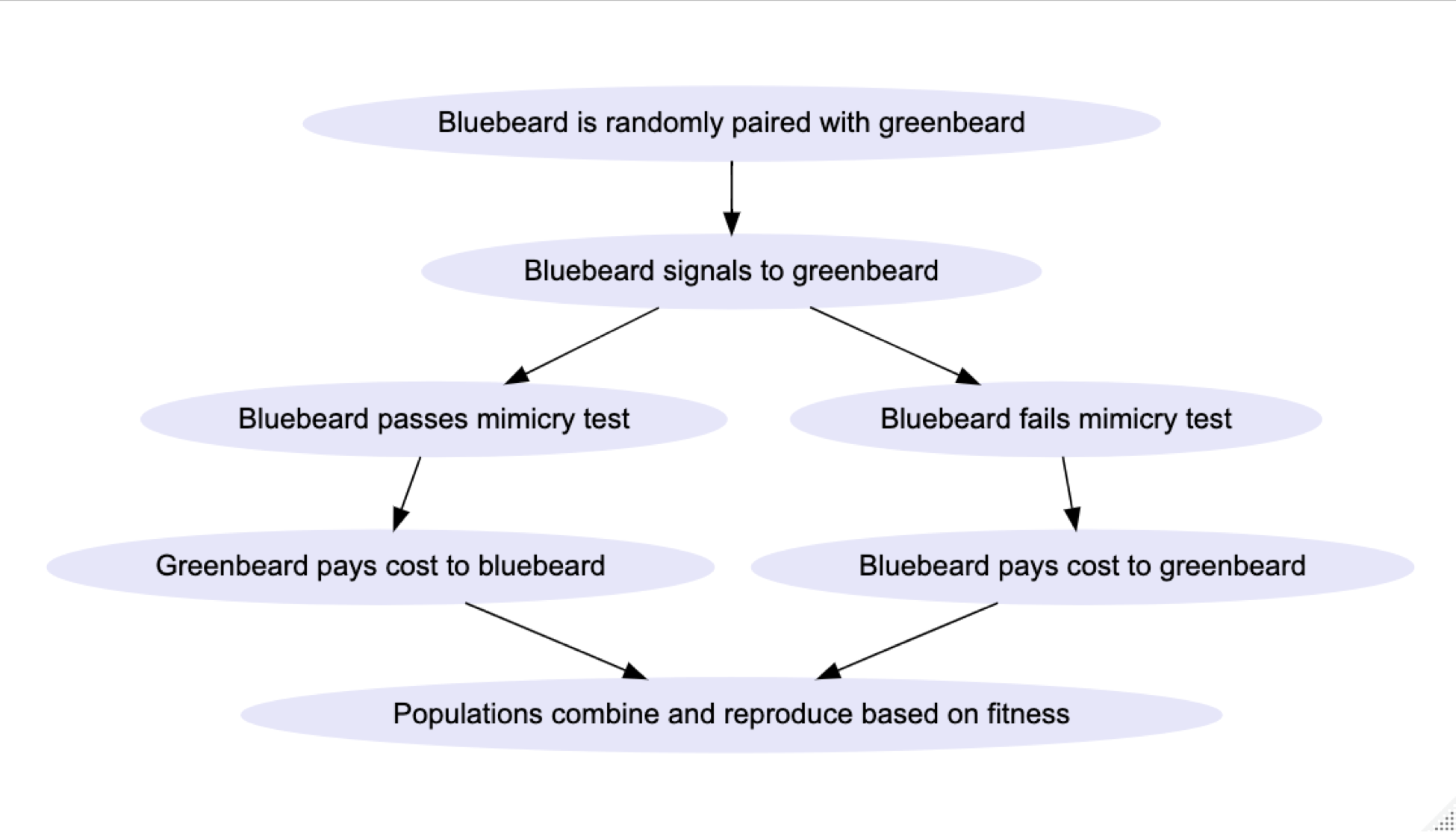

Supplement: S1 File — (ZIP) [file pone.0289333.s001.zip › Supporting-information/S1_Fig.tif]

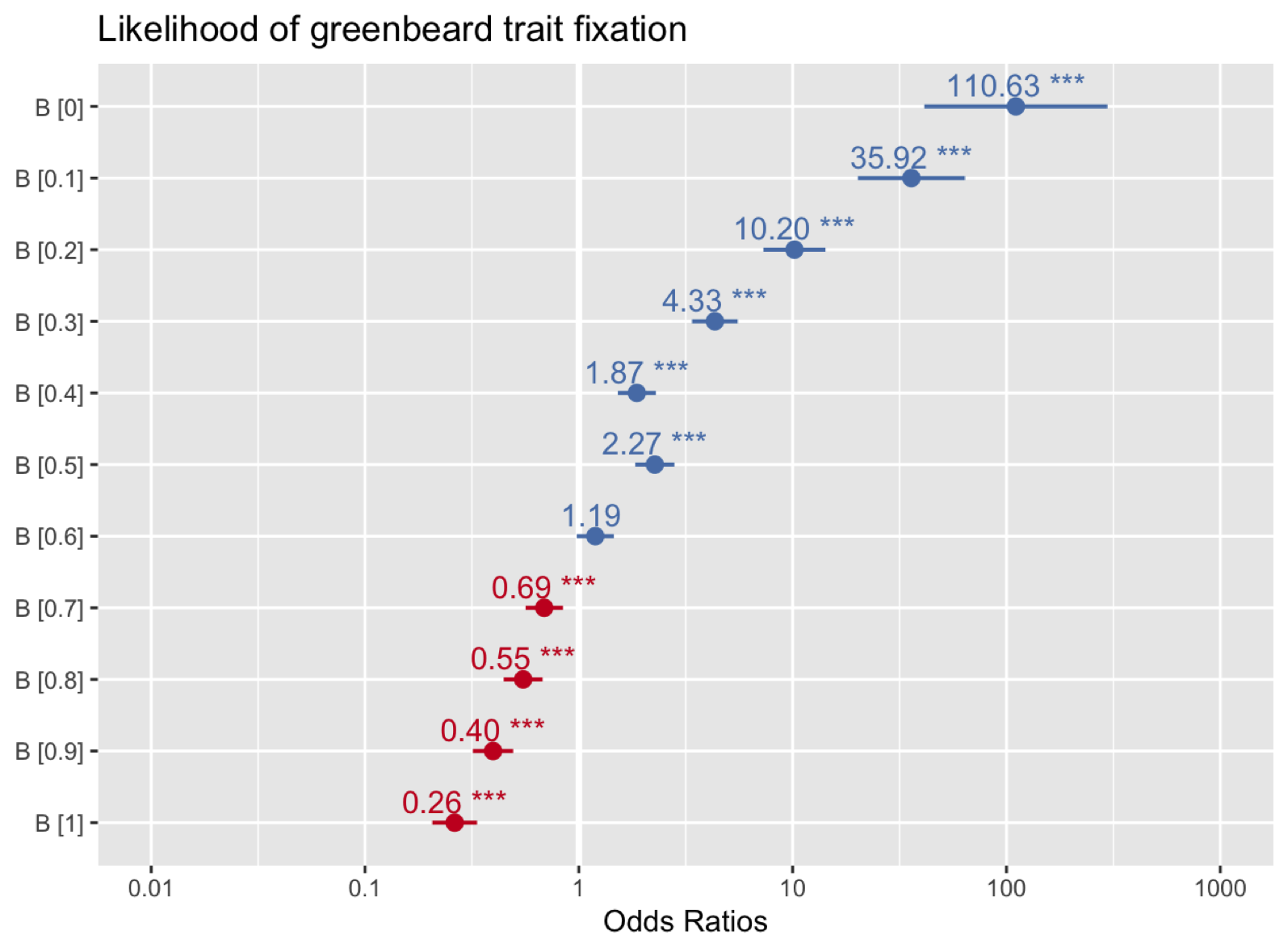

Supplement: S1 File — (ZIP) [file pone.0289333.s001.zip › Supporting-information/S8_Fig.tif]
