# Supplementary material for: Shibboleth: An agent-based model of signalling mimicry
Source: PLoS One. 2023 Jul 31;18(7):e0289333. doi: 10.1371/journal.pone.0289333 (PMC10389733; doi:10.1371/journal.pone.0289333)
Supplement: Attachment — Submitted filename: Response to reviewer.docx [file pone.0289333.s002.docx]

Dear Dr Blythe,

Thank you very much for sending us the reviewer’s comments, which we have found extremely helpful. And we apologize for the delay in sending our response — the edits required have been both computationally demanding and time consuming.

We would like to respond to each of the reviewer’s concerns in turn, and have given their comments in italics followed by our response in bold, and made changes where relevant in the manuscript and supplement.

Best wishes,

Jonathan R Goodman

*# Model specification*

*It is unclear what happens in the model at many places. There is supposed to be a standard protocol in the supplement, but at least in the online supplement (there was none attached with the manuscript) there is no such protocol. Either way, the main workings of the model should be described in the main text.*

**We have added a clearer standard protocol both in the supplement and the main text; see lines ~173-262. We have also added a table explaining the conceptual assumptions we make with justification in the relevant literature in Table S1.**

*I wonder whether the modelling approach is optimal. The authors have chosen to do an agent-based model. The advantages of such a model is that you can model basic mechanisms at the individual level, usually at the cost of transparency that more simple and broad-strokes models provide. However, most actions seem to concern the population level. There is heterogeneity in some parameter values and agents are paired in dyads, but the consequences concern the whole group. It seems to me that it would be possible to capture what is going on in a simpler population model. Alternatively, the model could be made truly agent-based, such that everything happens at the individual level, and if a dyadic interaction has consequences for more agents than those two involved, then that could be modelled explicitly. The model would then explain e.g. how other agents lose fitness if a certain agent fails to detect an outgroup individual, and population-level properties would emerge instead of being assumed. Currently, the model has the drawbacks of both population and agent-based models, without utilising their advantages.*

**We have developed two further versions of the model, and the Simple Shibboleth model is now explicitly an agent-based model without population-level effects, which we take to be a simpler, clearer model of the dynamics in which we are interested. This also allows for direct comparison between an entirely agent-based model and the model (now Models 3, 4, and 5) where there are population-level effects. The description and motivation for the new Model 1 is on lines 266-281 in the main text. We also explore the simple model more fully in the parameter space than the later models, primarily because of the demanding computational needs of running these models (see footnote 5 in the updated manuscript).**

*Line 233: Agents "might be reproduced in groups to which their parents did not belong". How? When does this happen? (And what does it reflect in reality?) Why are they assigned a random tolerance? What are the effects for the model outcomes?*

**We have clarified this by addressing a larger point that the reviewer refers to later, which is that we were previously agnostic about whether the traits in question were cultural or genetic. We believe that assuming the traits are cultural clears up a lot of ambiguities, which initially we thought were beneficial, but take the reviewer’s point that clearer assumptions help to interpret the models’ findings. We do note, however, that there may be a linkage disequilibrium between genetic and cultural traits in the respective groups. The description on lines 250-7 has been updated to address the reviewer’s problem noted in line 233 of the old manuscript.**

**Tolerance is randomly assigned, as with mimicry in the bluebeard population, to show within-group heterogeneity in the quality of mimicry detection, the boundaries of which are determined by the global variable *B*, which we describe on lines 234-42 and justify in the introduction.**

*How are tolerance boundaries updated? How are traits copied?*

**The first question we address where the reviewer refers to line 221. In response to the second question, we assume traits are copied via cultural inheritance, though the inheritance system could theoretically be horizontal or oblique.**

*Line 279: Explain what a "Fisher–Wright equivalent 'drift' model" means, in particular in this case.*

**See lines 330-334 in the updated manuscript. We mean that the model does not rely on any cultural selection bias, and a cultural trait should go into fixation only by chance in a given run.**

\## Minor remarks

*It is a bit curious that the model is described as being specifically about first allophones on a scale and then about colours on the spectrum from blue to green, the latter even to the extent of specifying R colour packages. It is fine to provide these as examples of what the numbers representing group membership can represent, but it should be clear that the model is about numbers on an ordinal scale.*

**See lines 144-52 in the updated manuscript.**

*It seems the simulations are not run for sufficiently many iterations. E.g. on line 313, 14% of the runs are unresolved. Could this not be solved just by increasing the number of iterations?*

**Yes, but we are also interested in whether adding a cultural selection mechanism (ie, cost-paying on failure to reproduce a cultural trait) reduces the likelihood that any cultural trait will go into fixation in the population.**

\# Model assumptions

*There are many specific modelling assumptions that seems more or less arbitrary. Many of these are probably important drivers of the results, so it is important to understand the rationale for the assumptions, and their effects.*

*A central assumption is that the tolerance of deviation and potential of mimicry use the same variable. Why is this? In reality, I would rather have guessed that these would be anticorrelated. The better the other group is at mimicking my phenotype, the more sensitive I should be to accept only those that are really close to my phenotype, rather than the opposite. This also seems to be a main driver of the results. If both tolerance of deviation and potential of mimicry are small, then greenbeards will do well, and at the other end, bluebeards will win. What do we learn from this?*

**The B variable is a central interest of ours, and as far as we know this is the first paper to explicitly use a variable that couples mimicry and mimicry detection. We justify this further in the introduction and methods in the updated manuscript — but the central consideration is that it is not possible to view mimicry and mimicry detection as independent qualities. A mimicry is only as good as the receiver is poor a detector, and therefore decoupling these qualities conceptually is impossible. The B variable accounts for this impossibility.**

**It may be tempting to view our findings as trivial given the design of the B variable. However, we note that in the psychological and evolutionary social sciences literature there is no real exploration of whether mimicry/mimicry detection is more important in a mimicry system than is the cost of being detected. We place this in perspective in the general discussion: the focus in work on cheater detection should place greater emphasis on probability of detection, rather than cost. Cost, in fact, seems largely irrelevant in this model, even, surprisingly, at very low values of B, which we believe is a finding with some important consequences for research in the evolutionary social sciences exploring cheater detection mechanisms.**

*Another coupling is that the cost when a greenbeard accepts a bluebeard is the same as it is for a bluebeard to be detected. What is the rationale for this? I would expect greenbeards to have to pay to sanction the bluebeard, but, on the contrary, now the cost comes when they do not sanction. Given the symmetry in costs, it seems just to moderate the size of the effect when bluebeards are or are not detected, and it comes as no surprise that varying the cost variable does not change the results qualitatively. But does this really model cost of sanctioning?*

**We take this point, and developed a new Model 2, a fully agent-based model, like Model 1, but where cost is decoupled for the agents. See lines 285-291 of the updated manuscript. In Model 1, we do not assume that sanction is costly, as there is no reason to assume it would be so in the classical Shibboleth story. We do, however, think it is worth exploring a situation where greenbeards pay no cost to help bluebeards, and have aimed to do this in Model 2.**

*Line 221: "Where bluebeards make up less than half of the population, boundaries increases the global tolerance boundaries range by (traits-1)*B". Why? What is the rationale for this and why is there a sharp cut-off? Why is this directional? And how is it implemented? This might have large consequences.*

**The rationale for updating tolerance boundaries is based on the assumption that, where there are a greater relative number of mimics in a population, it is likely that receivers are more likely to effectively detect mimics (and we further expect the reverse to be true), appealing to principles from work on Batesian mimicry (see lines 234-42). Using 50% as a sharp cutoff keeps the mechanism simple; we assume only that where the majority of a population are mimics, detection (relative to mimicry) will improve, and where the majority of the population are greenbeards, mimicry (relative to detection) will improve. Updating the variable globally also allows us to maintain interindividual variance.**

*Line 230: Where does the extra fitness cost for being a discerning receiver or more precise mimic come from (in reality)? Could this be modelled specifically instead of as a general cost?*

**We assume that there is a fitness cost to being a better mimic or detector. We note this in the supplement (Table S1); this is grounded in Zahavi’s foundational work on the handicap principle.**

*Figure 2: Why does everyone in the group lose PTR when one individual accepts a bluebeard, or a bluebeard is detected? This supposedly has far-reaching consequences for the outcomes. Why does every individual agent pay a tolerance score cost when a bluebeard has passed the mimicry test? Either the score is a cost for the agent paired with the bluebeard agents, but then only that individual should pay, or it is an investment cost to have such a detection system, but then that cost should not be contingent on a bluebeard agent having passed the test (with another agent).*

**We take this point, which we believe also relates to the notion above, which motivated us to redesign Model 1 as an agent-based model only. We retain the population-level effects in Models 3, 4, and 5; these indicate that the population-level effects make a large difference to the outcome. We explain the motivations for these effects fully in the updated manuscript (lines 295-305).**

*Model 2: Why is it interesting to study the case where agents have a higher chance of adopting traits 6 and 7? Why are they specifically five times more likely to adopt those traits? Why do they pay twice as high a cost (i.e. why is it higher, and why specifically twice)? And why are both changes made to the model at once?*

**Here (now Model 4), we were interested in understanding whether assigning a dual advantage to bluebeards — first, that greenbeards are more likely to have ‘blue-ish’ green cultural traits, and second, that the cost of a bluebeard entering the population has a larger effect on the green trait–pool than that assumed in Models 1-3 — would have a significant effect on the model’s outputs. We chose 5 because this ensured that while most greenbeards had non-extreme (ie, ‘blue-ish’) green traits, around 10-20% of greenbeards would retain an extreme green trait where N = 50. This accords with the idea that linguistic social and functional selection (see Nettle, 1999) will be weaker in the absence of strong cultural boundaries. See footnote 7 in the main document.**

*Model 3: "a low-tolerance greenbeard paid a fitness cost to reduce the high-tolerance greenbeard's tolerance to 0" and "Cost was twice the cost variable". Why would anyone do this in reality? Why is it reduced all the way to 0? And how is the “altruistic punishment” cost decided?*

**We assume that intolerant members of the greenbeard population will pay a cost to retain in-group homogeneity, which is foundational to cultural group selection theory. We chose twice the usual cost value to emphasize the importance of within-group enforcement mechanisms; we reduced tolerance to 0 to indicate that, following cultural group selection theory, a subset of in-group members are likely to pay a significant cost to maintain within-group homogeneity in cultural traits. Again we address this in footnote 7.**

\## Minor remarks

*The authors "remain agnostic as to whether the traits are cultural or genetic", but this should have some consequences for the model, e.g. as to whether agents can change their traits.*

**See the above point.**

*Why are "boundaries" proportional to "traits-1" instead of just "traits"?*

**See footnote 1 in the main text; this is a result of the peculiarities of the R language.**

*Figure 2: Should it be cost/10-tolerance, instead of cost/10+tolerance? The cost increases the less tolerant the agent is. And why is cost divided by 10, instead of just using values of the parameter that are ten times smaller than now?*

**Yes, we have corrected this.**

\# Model interpretation

Related to the assumptions, it is often hard to interpret what the results of the model mean. Overall, I wonder if the right thing is being measured.

*I wonder if it is correct to see the greenbeard phenomenon as a situation of competition between groups. You want to exclude freeriders from benefitting from cooperative norms in your group, not to compete with another group. It is not modelled here what is the difference between the greenbeards and the bluebeards, only that bluebeards want to mimic the greenbeards. It seems in the model that agents (or their offspring) can change groups. What does it mean then, when a group has gone into fixation? If everyone has adopted a green trait, is that really a success for the greenbeards, if the purpose is usually to keep the others out? Most of the analyses are about which group has taken over the entire population, but I wonder if this is really the right measure, and how to interpret such a result. Had the model been explicit about what is at play, and modelled within-group interactions explicitly, then it would have been possible to measure e.g. whether cooperation is maintained within the greenbeards, or if benefits from cooperation outweigh costs of trying to keep bluebeards out (either through detection or through imposing higher costs), which is what I think the authors are actually after.*

**We take as a premise that the groups practice parochial altruism, and moreover that individuals who share cultural traits are more likely to behave altruistically towards one another (Table S1). We are therefore interested in which cultural traits go into fixation, not whether these groups maintain high rates of cooperation, which we believe other models and lab experiments have shown effectively in previous work.**

**We have attempted to explain more fully our explicit interest in the traits themselves in the introduction and methods. We are interested only in the effects of likelihood of detection vs cost of being detected; the within-group interactions are assumed to be irrelevant to this (as, we believe, the likelihood of detection is not considered in many papers evaluating parochial altruism or cooperation dynamics).**

*A main result is that detection is more important than costs. But this seems to be fundamentally built into the assumptions, as described above. Acceptance is coupled positively(!) with the potential of the outgroup to mimic, and costs are applied equally to both sides. I would be hesitant to make interpretations of this to apply to detection and sanctioning frauds in general. Line 462: “we found that B … was an excellent predictor”, but it is designed so that low values favour green and high values favour blue.*

**See the above re the reviewer’s point about coupling mimicry and mimicry detection. We believe the distinction between this coupled quality, and the cost of detection, is an essential one that previous papers do not consider in detail, and our aim here is to draw attention to the importance of likelihood of detection, as represented by the B variable.**

\## Minor remarks

*Some tables and figures are missing, or have the wrong numbers, e.g. Figure S1 (or Figure 7 on line 456 or Figure 6 on line 466) is not what is decribed on line 168, and there are no tables in the supplement (or a Figure 19, line 550).*

*There are many figures that seems to say very similar things. I think the number of figures could be reduced considerably and be more to the point.*

**We have addressed both points in the main text, and reduced the number of figures.**

\# Literature review

*As I said above, the literature review is generally well written, pertaining to the background on groups and cooperation. However, there should probably be more focus on references to explain and motivate the model assumptions. I also have a few suggestions for references that seem relevant for the background:*

*## Parochial altruism and tag-based cooperation*

*- Fu, F., Tarnita, C. E., Christakis, N. A., Wang, L., Rand, D. G., & Nowak, M. A. (2012). Evolution of in-group favoritism. Scientific Reports, 2, 1–6. https://doi.org/10.1038/srep00460*

*- Jansson, F. (2015). What games support the evolution of an ingroup bias? Journal of Theoretical Biology, 373, 100–110. https://doi.org/10.1016/j.jtbi.2015.03.008*

*## Greenbeards and language*

*- Lindenfors, P. (2013). The green beards of language. Ecology and Evolution, 3(4), 1104–1112. https://doi.org/10.1002/ECE3.506*

*- Jansen, V. A. A., & van Baalen, M. (2006). Altruism through beard chromodynamics. Nature, 440(7084), 663–666. https://doi.org/10.1038/nature04387*

*- Cohen, E. (2012). The Evolution of Tag-Based Cooperation in Humans. Current Anthropology, 53(5), 588–616. https://doi.org/10.1086/667654*

**We thank the reviewer for these references, some of which we have added to the paper and supplement. We note that Cohen’s 2012 paper was closely considered in the initial submission, and motivated several of the assumptions in this paper.**

\## Population structure

*I do not think that not assuming a lattice structure is something that needs to be motivated. There are problems with such assumptions, and alternative approaches might be better, as illustrated in these papers, in case the authors still want to keep such a motivation in the manuscript:*

*- Jansson, F. (2013). Pitfalls in Spatial Modelling of Ethnocentrism: A Simulation Analysis of the Model of Hammond and Axelrod. Journal of Artificial Societies and Social Simulation, 16(3), 2. http://jasss.soc.surrey.ac.uk/16/3/2.html*

*- Read, D. (2010). Agent-based and multi-agent simulations: coming of age or in search of an identity? Computational and Mathematical Organization Theory, 16(4), 329–347. https://doi.org/10.1007/s10588-010-9067-1*

*- Tarnita, C. E., Antal, T., Ohtsuki, H., & Nowak, M. A. (2009). Evolutionary dynamics in set structured populations. PNAS, 106(21), 8601–8604. https://doi.org/10.1073/pnas.0903019106*

**We have removed reference to lattice structures.**
