# Supplementary material for: Shibboleth: An agent-based model of signalling mimicry
Source: PLoS One. 2023 Jul 31;18(7):e0289333. doi: 10.1371/journal.pone.0289333 (PMC10389733; doi:10.1371/journal.pone.0289333)
Supplement: Attachment — Submitted filename: Response to reviewer.docx [file pone.0289333.s004.docx]

**Responses to referee**

**Referee’s comments in italics, responses in bold**

*What maintains linguistic diversity? This theoretical study investigates one mechanism, which is the association of particular linguistic variants with parochial altruism. This is in part inspired by the Biblical story of Shibboleth, where a particular pronunciation of the word Shibboleth is used as a marker of in-group, out-group status. The authors construct an agent based simulation to consider the scenario when two populations meet (“Greenbeards” and “Bluebeards”), and consider how differing degrees of tolerance, mimicry, as well as costs and benefits to these actions (as well as failure to detect or mimic) may alter the effects of parochial altruism on the frequency of different linguistic phenotypes.

I think the question that the authors investigate is an interesting one, although I have questions about why they chose the particular modelling approach that they did (with many of the same queries as the previous reviewer), where perhaps a simpler modelling approach would have been clearer to understand and generated many of the same behaviours. For example, why simply not simply two variants with probabilities of acceptance/rejection (rather than the 10 variants that are used), or why not have a symmetrical interaction structure where individuals can interact with anyone in the population, then you would be able to investigate potentially other types of signalling error (i.e. rejecting someone who is actually of the same type). I think some of this would appear more natural (at least to me!) than the setup investigated.*

**We used ten variants to highlight the spectrum between s and esh on a phonemic continuum, and to evaluate how the processes we are interested in might lead to non-extreme traits (on the spectrum) might be adopted. We do not evaluate this here, but believe this would be an interesting question for future versions of this model evaluating cultural change.**

**We also did not evaluate a population with a symmetrical structure for the sake of simplicity, especially in later models where the dynamics were more complex. Realistically, we would (of course) assume that signalling errors are possible, and believe this would be an interesting dynamic to investigate further. We have made amendments throughout the paper to reflect these points (these amendments are noted specifically below).**

*Nonetheless, the authors have responded in a substantial manner to the previous review, and so I think many of the issues I have can be dealt with by a simple clarification of the methods. I also think the authors have done an excellent job of making their code associated with their simulations accessible, so that others in the future will also be able to probe and build upon their models in the future.*

Main points

*The major point that I still do not think is very clear is the underlying demography and inheritance structure. As far I can see we begin with an equal number of “Greenbeards” and “Bluebeards”, and then individuals reproduce according to their fitnesses (which arise from a combination of the costs and benefits they experience during the social interactions). This presumably means that it likely that in the next generation there will be an unequal number of “Greenbeards” and “Bluebeards”. Does this that the less frequent groups have individuals that pay twice the penalty/get twice the benefits from the social interactions? Or does it mean certain individuals do not undergo the social interaction? For example, what does it mean “Where traits were reproduced into the group to which their cultural parents did not belong (L255-256)”. I don’t really think this aspect is described, and so again I think more explanation is required to outline how this aspect of the demography functions.*

**We take this point, and recognize the manuscript did not effectively describe some of these population dynamics. When the model initiates, the two populations are separate, and the “bluebeards” enter the “greenbeard” population in a dynamic resembling the Shibboleth story. Thereafter, however, the greenbeard and bluebeard groups join into a single population, and agents reproduce from that population of 100 individuals with probability *fitness* (with the possibility for some individuals to replace multiple times).**

**Therefore, in generation 2 and onwards, the dynamic is the same as in generation 1 but the population is mixed, and individuals who were reproduced by an ancestral greenbeard may interact with another greenbeard in the same way a bluebeard did in generation 1 (this is the point of L255-256 in our prior submission). We have amended this section to make the dynamics clearer. But the critical point is that the cultural trait borne by an individual is what defines them as a greenbeard or bluebeard, and any dyadic interaction (bluebeard/bluebeard, bluebeard/greenbeard, and greenbeard/greenbeard) is possible. See line 255-258, where we aimed to make this clear.**

*Related to this it is unclear to me both some of inheritance of certain traits, or why certain choices about what is heritable/what is not were made. This is important, because of course the choices about this govern what aspects of the system are evolving. It appears that individuals simply inherit the trait of their parent with perfect fidelity. Is this correct? If so, that presumably means the only source of linguistic variation is that which the population started with (i.e. there is no equivalent of mutation in the model). Similarly, I was confused about what traits were heritable (and therefore potentially subject to evolutionary pressures in the model). For example, parent “fitness” seems to be. Does this mean that individuals themselves do not start form a blank slate, but instead inherit the fitness state of their parents? If so, this seems an unconventional choice, and I am not sure what it adds to the model. Similarly, it seems like mimicry was heritable. So again, we would expect the mimicry values to change throughout the course of the model, but as far as I can see this is not commented on? Again, it seems odd to assume mimicry is heritable but tolerance is not (except in Model 5). Again here the evolutionary dynamics of toleration is not commented upon (as far as I can see). [Indeed, I think the authors could make clearer from the jump what aspects of their system they are investigating the evolution of, and which are simply random variables each generation.]*

**We have rewritten the paragraph starting at line 251 to make the inheritance structure in the overall population clearer. We wrote the functions so that offspring inherit both fitness and trait with perfect fidelity only to keep the model as simple as possible — we were interested only in the effect of the Shibboleth interaction on the distribution of cultural traits in the population. Realistically we would of course assume fluctuation/mutation in these characteristics, and later models could evaluate the impact of such dynamics.**

**Mimicry was partially heritable in models 1-4 for the same reasons, though tolerance could not be inherited because the tolerance boundaries were updated at each generation, while mimicry boundaries were not — this was a consequence of the receiving population adjusting tolerance based on the proportion of mimics in the population, which we justify in the manuscript on lines 235-243.**

**Furthermore, the random draw of tolerance at every new generation functioned as the primary stochastic element of the model dynamic. Preserving fidelity in the other individual-level qualities allowed us to investigate the impact of updating tolerance boundaries using the value of B without the noise of other random variation. Again, adding random mutation in would be interesting to explore in the future, but for the sake of simplicity and isolating our dynamic of interest we did not do so here. See footnotes 3 and 4 in the manuscript where we aimed to address these points.**

*The central finding, which the authors focus on, is the fact that B, which then scales the range outside one’s focal value with which one is tolerant, and scales the range outside which one can mimic, has a strong effect on whether the detector/mimic increases in frequency. Maybe unsurprisingly, when individuals can mimic a greater range of values they increase in frequency (“Bluebeards” more likely to go fixation), and when they cannot they don’t (“Greenbeards” more likely to go fixation). They find that this pattern holds across many of their simulations. As they point out in their previous response to reviewers this might seem “trivial”, although I don’t see that as an issue, one would hope that behaviours of a model that one expects to see do actually emerge in the outcomes of the model. Moreover, one might expect it to interact more strongly with costs, although this is hard to see outside of Model 1 as they typically do not vary both factors simultaneously.

In contrast to B, across their models, they find that the absolute various costs imposed have little qualitative effect, although as their model 2 shows, potential asymmetries in costs may change the outcome qualitatively.*

*As an aside, it is a shame that for all the runs the authors weren’t able to keep the simulation going until fixation. Because one of the interesting aspects of the model would be not only to look at whether green/blue was more likely to fix, but the average time to fixation. This would be a nice proxy for how well this particular system might be able to maintain heterogeneity of signals. The authors do mention that in their Model 1 fewer runs went to fixation than in the drift scenario, and this would be an interesting angle to develop, and I wonder whether they noticed any other patterns determining the proportion of runs that went to fixation in the population. Alternatively, they could look at another measure of signal diversity in their populations through time (maybe they could look at something equivalent to heterozygosity) and investigate the rate at which it declines. Maybe here factors like costs do have an impact affect outcome.*

**These are interesting dynamics, and ones we plan to explore in the future. Here we did not run all simulations to fixation only because the model was so demanding computationally, and given that a large majority of runs went into fixation by generation 200, we decided to cap the runs at that number. Similarly, we did not explore diversity specifically because, while interesting, it was not our primary focus, though we recognize that this would be something to explore in the future. We have amended footnote 7 to reflect this.**

*Finally, I do wonder whether to streamline the paper, more of the focus could be placed on Model 1, as this is the simplest, most thoroughly investigated, and captures much of the behaviour that the authors discuss, with the other models made a little more ancillary (or even moved into the Supplementary Material). But this is purely a personal preference.*

**We recognize this point, but would like to keep the subsequent models in the paper. The primary focus — of both the analysis and discussion — is on Model 1, but that the other models add important aspects that are subsidiary to Model 1. Specifically, introducing population-level effects as well as punishment seems to have a dramatic impact, which we feel warrants discussion in the main text.**

Minor points

*The referencing between colours/phonemes feels a bit unnecessary. Ultimately, these are all just illustrations of it being a number in the actual mechanics of the model. Whilst having the illustration helps, flitting between them or adding in oddly specific details (e.g. “Specifically, we assume for convenience that there are ten linguistic variables between esh (ʃ; voiceless palato-alveolar fricative) and s (unvoiced alveolar sibilant), which we represent on an ordinal scale with a range of 1-10, and visualise on a colour spectrum between green (esh) and blue (s).”) which frankly have little to do with the actual model they have created and are just a little distracting. I would suggest slimming down and focusing on the colour element (although this is more a personal choice of what I think is easier for the reader to follow).*

**We have updated the language to focus more on the colour spectrum.**

*Many of the parameters in the model, do seem chosen a little arbitrarily, and it’s not clear what effect they have on the results. For example, in the population cost model everyone in the population pays the costs for the success/failure of the interaction, why was 10 chosen as the factor? Or indeed why were 10 phonemes chosen in the first place? I understand that with a model such as this there will be aspects that simply need to be chosen, but then I think this speaks to the strengths of a simpler model, with fewer parameters, whose behaviour can be more thoroughly investigated.*

**We chose 10 as a factor in the population-level effect models because this was the value that appeared, when we were first formulating the model, the most likely to exert any effect without the effect being large enough to rival those resulting from the dyadic interactions. We also chose 10 phonemes as this appeared to be the number of realistically distinguishable linguistic variants between esh and s. More broadly, we appreciate both these points, and the need to choose values for these variables further necessitated removing noise in the rest of the model dynamics, discussed above. The paragraph beginning on line 673 aims to address this.**

*L22 - “Here we describe the results of three agent-based models that simulate multi-generational interactions between two groups of individuals” – I thought it was 5?*

**It is 5 — thank you for noting this.**
